# Insulin Sensitization by PPARγ and GLUT-4 Overexpression/Translocation Mediates the Antidiabetic Effect of *Plantago australis* †

**DOI:** 10.3390/ph16040535

**Published:** 2023-04-03

**Authors:** Samuel Estrada-Soto, Kathia Ornelas-Mendoza, Gabriel Navarrete-Vázquez, Fabiola Chávez-Silva, Julio Cesar Almanza-Pérez, Rafael Villalobos-Molina, Erandi Ortiz-Barragán, Hilda Loza-Rodríguez, Julio César Rivera-Leyva, Angélica Flores-Flores, Irene Perea-Arango, Javier-German Rodríguez-Carpena, Gabriela Ávila-Villarreal

**Affiliations:** 1 Facultad de Farmacia, Universidad Autónoma del Estado de Morelos, Cuernavaca 62209, Mexico; 2 Laboratorio de Farmacología, Depto. Ciencias de la Salud, D.C.B.S., Universidad Autónoma Metropolitana—Iztapalapa, Ciudad de Mexico 09340, Mexico; 3 Unidad de Biomedicina, Facultad de Estudios Superiores Iztacala, Universidad Nacional Autónoma de México, Tlalnepantla 54090, Mexico; 4 Laboratorio de Microbiología Experimental, División de Tecnología Ambiental, Universidad Tecnológica de Nezahualcoyotl, Nezahualcoyotl 57000, Mexico; 5 Departamento de Inmunofarmacología, Instituto Nacional de Enfermedades Respiratorias, Ciudad de Mexico 14080, Mexico; 6 Centro de Investigación en Biotecnología, Universidad Autónoma del Estado de Morelos, Cuernavaca 62209, Mexico; 7 Centro Nayarita de Innovación y Transferencia de Tecnología “Unidad especializada en I+D+i en Calidad de Alimentos y Productos Naturales”, Universidad Autónoma de Nayarit, Tepic 63000, Mexico; 8 Unidad Académica de Ciencias Químico Biológicas y Farmacéuticas, Universidad Autónoma de Nayarit, Tepic 63000, Mexico

**Keywords:** α-glucosidases inhibition, antihyperglycemic, GLUT-4, PPARγ, oral toxicity assays, ursolic acid, caffeic acid

## Abstract

*Plantago australis* Lam. Subsp. hirtella (Kunth) Rahn is a medicinal plant used as a diuretic, anti-inflammatory, antibacterial, throat cancer treatment and for the control of diabetes. *P. australis* was collected in the state of Morelos, México. The hydroalcoholic extract (HAE*Pa*) of *P. australis* was obtained by maceration and concentrated in vacuo. Once dry, it was evaluated through an oral glucose tolerance test (OGTT) in normoglycemic mice and in a non-insulin-dependent diabetic mice model. The expression of PPARγ and GLUT-4 mRNA was determined by rt-PCR, and GLUT-4 translocation was confirmed by confocal microscopy. The toxicological studies were conducted in accordance with the guidelines suggested by the OECD, sections 423 and 407, with some modifications. HAE*Pa* significantly decreased glycemia in OGTT curves, as well as in the experimental diabetes model compared to the vehicle group. In vitro tests showed that HAE*Pa* induced an α-glucosidase inhibition and increased PPARγ and GLUT-4 expression in cell culture. The LD_50_ of HAE*Pa* was greater than 2000 mg/kg, and sub-chronic toxicity studies revealed that 100 mg/kg/day for 28 days did not generate toxicity. Finally, LC-MS analysis led to the identification of verbascoside, caffeic acid and geniposidic acid, and phytochemical approaches allowed for the isolation of ursolic acid, which showed significant PPARγ overexpression and augmented GLUT-4 translocation. In conclusion, HAE*Pa* induced significant antidiabetic action by insulin sensitization through PPARγ/GLUT-4 overexpression.

## 1. Introduction

Diabetes is a chronic, non-transmissible, progressive metabolic disease characterized by hyperglycemia with metabolic disorders [1]. It is usually accompanied by inadequate insulin secretion, tissue resistance to its action or a combination of both [2]. This disease reduces the quality of life of patients and their families and increases the risk of death, and given the large number of complications that frequently occur, it also has an important impact on the economy of patients and health services [3]. According to the IDF, in 2021 there were 537 million people with diabetes, with 90% of cases corresponding to type 2 (T2D). One in two adults with diabetes is undiagnosed (232 million people), and 531 million people have impaired glucose tolerance. Under this scenario, it is estimated that 10% of global health spending goes to diabetes (close to USD one trillion). Currently, the treatment for T2D includes several pharmacological groups that are intended to maintain adequate glycemic control; however, poor physical activity, poor eating habits, and a lack of adherence have increased tolerance for conventional medications. In addition to this, the emergence of new diseases, such as COVID-19, which more frequently affect patients with diabetes and concomitant diseases, make it necessary to permanently search for new pharmacological treatments and alternatives aimed at the prevention and control of these pathologies [4].

México has a great botanical diversity. It is estimated that it has 10–12% of the world’s biodiversity (31,000 different species), which supports the use of a wide variety of plants for medicinal purposes [5]. In México, 3350 plants form a part of the medicinal flora, and from these 269 are employed empirically in the alternative treatment of diabetes, but there are only 80 species with scientific reports, and only in 50% of cases has their hypoglycemic effect been verified [5,6]. Therefore, *Plantago australis* Lam. (Plantaginaceae), known in México as “gusanillo” or “llantén”, is used for the treatment of disorders of the digestive system, to “deflate” the kidneys and lungs, and for throat disorders and cancer; for the treatment of diabetes, a decoction of aerial parts (mainly leaves) are used [7,8]. Additionally, in some countries, the leaf infusion of *P. australis* is often used as a laxative and a diuretic, for anti-inflammatory and antibacterial purposes, and for wound healing [9,10,11]. Among the pharmacological effects described for this species, the analgesic and anti-inflammatory potential of the ethanolic extracts of leaves, roots and fruits stand out [10].

Previous studies have also demonstrated that a standardized hydroalcoholic extract of *P. australis* (verbascoside, 6%) possesses anti-inflammatory and wound healing properties [12]. Regarding the chemical composition of the genus Plantago, phenylpropanoid glycosides, iridoids, triterpenes, flavonoids and phenolic acids have been found in the aerial parts, as well as polysaccharides in the seeds. The chemotaxonomic marker of this genus is glycosylated iridoid-type compounds [13], and for *P. australis*, aucubin, baicalein, oleanolic acid, ursolic acid, salidroside, isoverbascoside and verbascoside have been isolated [14]. Further, Nemitz et al. found compounds such as neolignins, cinnamic acid derivatives, flavonoid (luteolin 7-glycoside), and anthocyanic pigments [15]. The presence of these compounds suggests a significant antidiabetic action of the plant, with mechanisms of action related to α-glucosidase inhibition and/or insulin sensitization mainly due to the presence of UA, verbascoside and caffeic acid.

Currently, there are several groups of antidiabetic drugs available on the market. These drugs are classified based on their mechanism of action, which in general act as secretagogues, antihyperglycemics and/or insulin sensitizers. Within the latter, it can be mentioned that the nuclear Peroxisome Proliferator-Activated Receptors (PPARs) are the protein targets of many endogenous fatty acids, which act as anti-dyslipidemic and insulin-sensitizing compounds.

Moreover, PPARs regulate target genes involved in several biochemical pathways; for example, glucose transporter GLUT-4 [16] PPAR modulation decreases blood glucose in hyperglycemic individuals via an improvement in insulin sensitization, and the increase in GLUT-4 transporters in striated muscle is very important for glucose homeostasis.

Thus, the aim of the current investigation was to determine the antihyperglycemic and antidiabetic effects of *P. australis* and its possible mechanisms of action, and to identify the potential bioactive compounds responsible for its antidiabetic activity. Additionally, we aimed to provide approaches for its safety through acute and subacute toxicological assays.

## 2. Results and Discussion

### 2.1. Phytochemical Characterization

#### 2.1.1. Identification and Quantification of UA by HPLC

**Figure 1:**
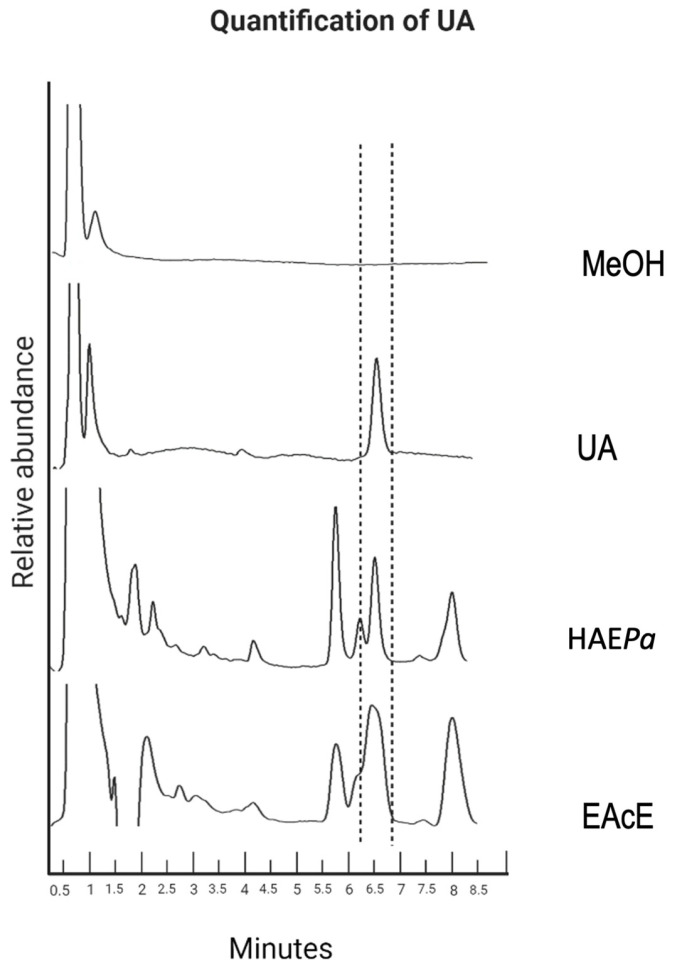
Overlayed HPLC profiles of ursolic acid present in HAE*Pa* and EAcE. Chromatographic conditions: Gemini column (4.6 mm × 75 mm); mobile phase: methanol/acidified water 85:15 (*v*/*v*), flow rate 0.9 mL/min, λ = 205 nm.

Throughout the maceration and extraction process of a hydroalcoholic extract of *Plantago australis* (HAE*Pa*) and ethyl acetate extract (EAcE), a white powder precipitate was obtained and s qualitatively identified as ursolic acid (UA) using thin layer chromatography (TLC), in comparison with a UA standard reference and LC-MS experiments. Additionally, 1D NMR spectra were acquired for the precipitate, to confirm the structure of ursolic acid (further details in Figures S1 and S2 in the Supplementary Material). After confirming its identity and structure, UA was quantified by HPLC, as shown in Figure 1, accounting for the fact that 49.6 mg of the extract contains 0.36 mg of UA (0.73% of UA/1 g of HAE*Pa,* or 7.26 µg/mg). The limit of detection (LOD) and limit of quantitation (LOQ) values for UA were 0.21 µg/mL and 0.63 µg/mL, respectively, at λ = 205 nm [17].

Triterpenic acids, such as oleanolic and ursolic acids, were formerly isolated from *P. australis* [14], which could be related to the antidiabetic effect mentioned in folk medicine [18].

#### 2.1.2. UPLC-ESI-MS Strategy for Compounds Identification

Contrasting with the single chemical entity that usually is the basis of modern pharmacology and drug development, the challenge of traditional herbal medicine is the multi-compounds in herbal preparations. Chemical fingerprint plays an important role in the assessment of complex analytes. Herbal drugs, singularly and in combination, contain a myriad of compounds in complex matrices in which frequently no single active constituent is responsible for the overall efficacy. This creates a challenge in establishing a strategy for profiling chemical components. This difficulty has been acknowledged in the draft of a Strategic Plan for Regional Traditional Medicine of the World Health Organization (WHO) [19].

As a preliminary assessment to develop a chemical fingerprint for *Plantago australis,* the HAE*Pa* and the EAcE precipitate were analyzed by HPLC, and UA was identified. Afterward, HAE*Pa* and EAcE were subjected to a preliminary phytochemical characterization using UPLC-ESI-MS with single quadrupole mass spectrometry (QDa). A full mass scan (50–1250 Da) and a targeted Selected Ion Recording (SIR) experiment were performed for targeted compounds identification based on chemotaxonomic markers [20,21,22] for the Plantaginaceae family and the preliminary result from TLC (Figure S1).

**Figure 2:**
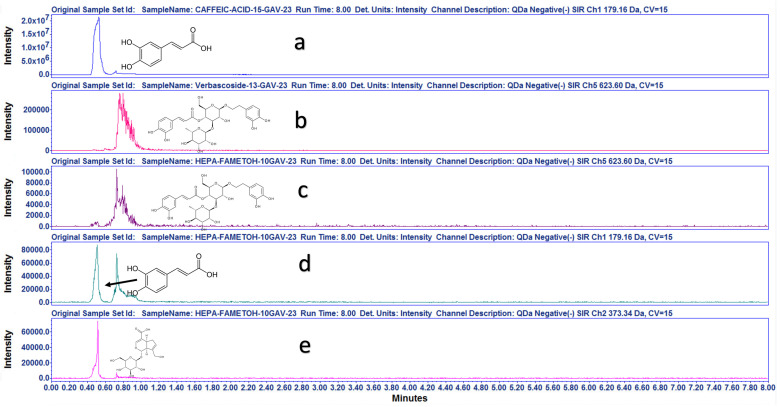
Stack plot from SIR channels for standard compounds and HAE*Pa*. Negative-mode SIR experiments for identification of caffeic acid 179.16 Da, verbascoside 623.60 Da, and geniposidic acid 373.34 Da. Standard compounds (**a**,**b**), HAE*Pa* (**c**–**e**).

A targeted negative-ion-mode SIR experiment was conducted for verbascoside [623.60, M-1] (Figure 2b), caffeic acid [179.16, M-1] (Figure 2c), and geniposidic acid [373.34, M-1] (Figure 2d). HAE*Pa* was compared with internal standards of verbascoside and caffeic acid previously identified by NMR experiments. All the above experimental results verified the presence of verbascoside, caffeic acid and geniposidic acid, an iridoid broadly distributed in the *Plantago* species extract [20].

**Figure 3:**
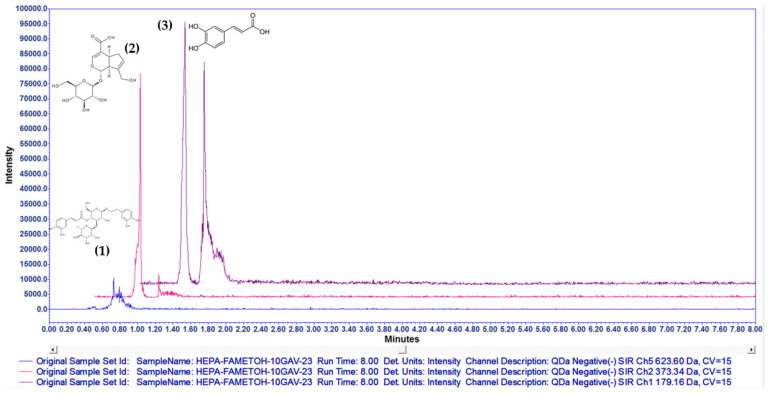
Overlayed chromatograms of SIR experiments for each compound present in HAE*Pa* in the same plot (1) verbascoside (2) geniposidic acid (3) caffeic acid.

Additionally, to survey the compounds in the hydroalcoholic extract, the chromatogram was aligned with and without standards, as shown in Figure 2 and Figure 3. In Figure 3, the major chromatographic peak labelled as number 3 is for caffeic acid, the second major peak (number 2) is for geniposidic acid, and the minor peak (number 1) is for verbascoside. These compounds have shown significant antihyperglycemic and antidiabetic activities [12,23,24].

**Figure 4:**
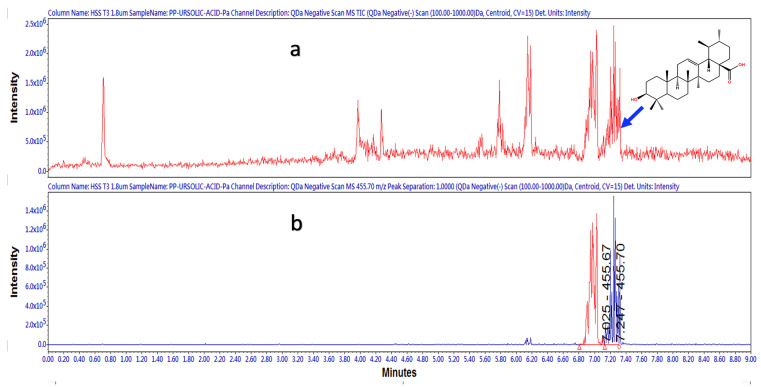
(**a**) EAcE total-ion chromatogram mass scan from 100 to 1000 Da in negative mode (**b**) SIR channel for m/z 455.70 in negative mode.

**Figure 5:**
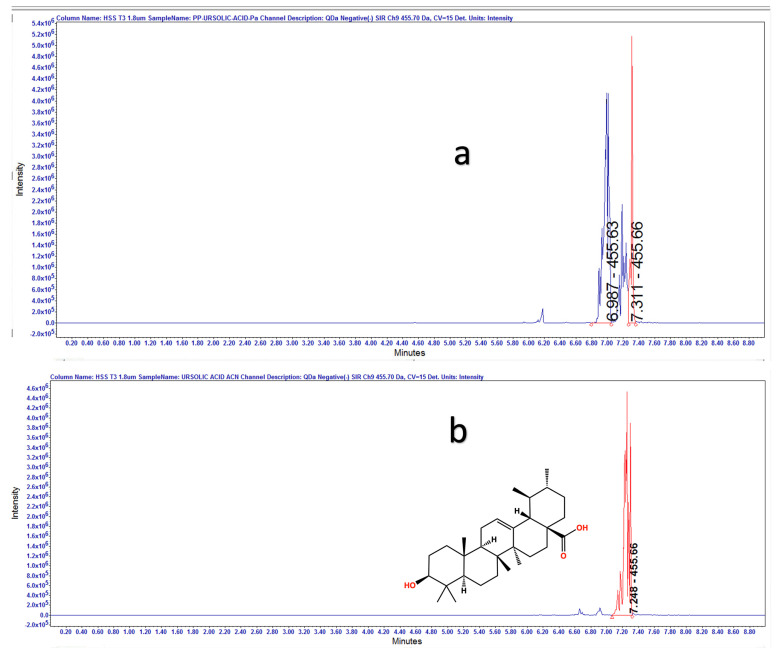
SIR experiments for EAcE (**a**) and UA commercial standard (**b**), channel selected 455.7 Da in negative mode with a retention time at 7.248 min.

The complexity of the HAE*Pa* sample did not allow us to adequately observe the presence of UA even though it was evident from TLC and HPLC, as previously described. To corroborate the presence of UA, EAcE was analyzed using LC-MS in negative-ion mode. Figure 4 shows the Total Ion Chromatogram (TIC) for EAcE with a retention time (RT) of 7.247 min and a mass of 455.70 [M-1]. In Figure 5, we can observe the positive identification of UA in EAcE compared with the commercial standard, with a retention time of 7.311 min and 7.248 min, respectively. In Figure 5a, there is a peak at 6.987 min and another one at 7.311 min for UA. Oleanolic acid (OA) and UA are isomeric triterpenic acids and are broadly distributed as mixtures in plants. The only difference between the two isomers is the position of one methyl. OA and UA always exist in the same plant, so it is difficult to separate them [25]. OA has previously been identified in *Plantago australis,* and tentatively the peak at 6.987 min (Figure 5a) is assigned as OA [26,27].

### 2.2. In Vivo Pharmacological Studies

As described in Section 1, *Plantago australis* is a medicinal plant used in traditional medicine as an antidiabetic agent. This study analyzed a hydroalcoholic extract to determine its antidiabetic activity. There is a lack of scientific evidence regarding the antidiabetic properties. Furthermore, to ensure safety in its use, acute and subacute toxicity was evaluated based on the guidelines that are suggested by the OECD, with some modifications. With the results obtained from the pharmacological and toxicological activities, and once the extract is standardized and the preclinical studies are carried out, it could be proposed as a potential phytomedicine for the treatment of T2D.

#### 2.2.1. Oral Glucose Tolerance Tests

**Figure 6:**
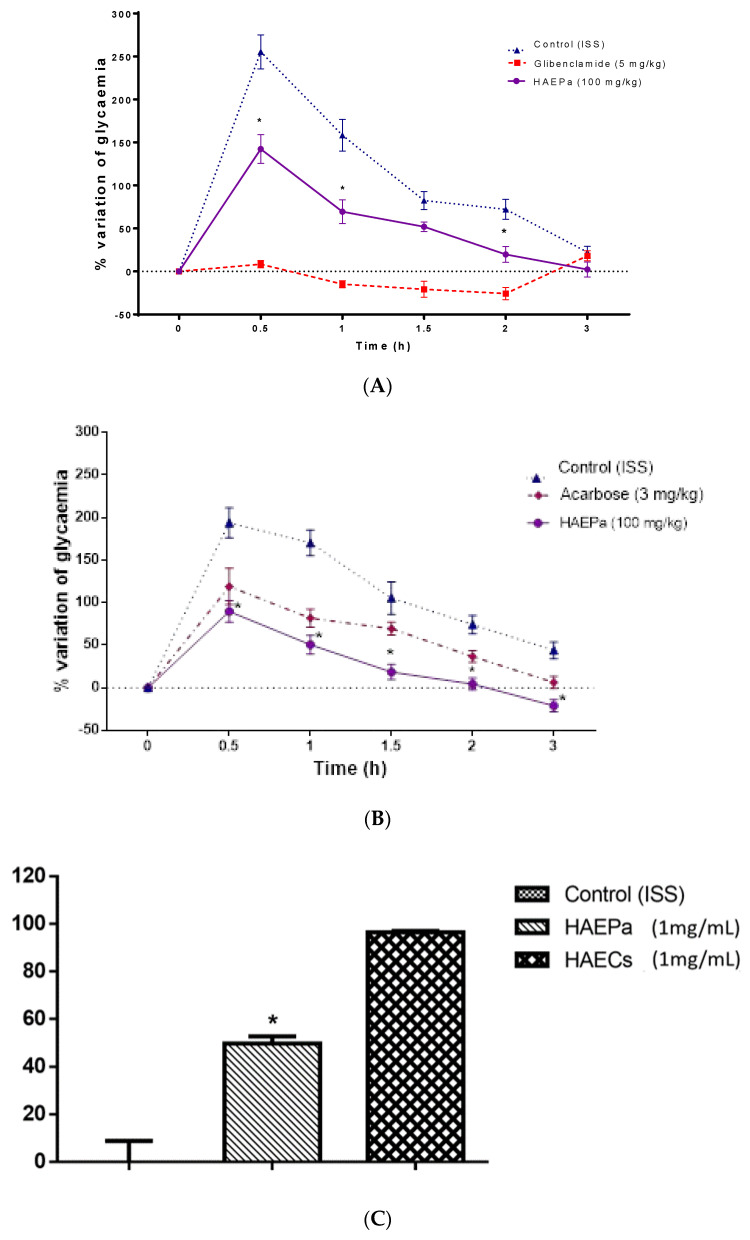
In vivo experimental oral glucose tolerance tests: (**A**) Effect of hydroalcoholic extract of *Plantago australis* (HAE*Pa*) on glycemia after a single oral administration of 2 g/kg of glucose in male normoglycemic CD1 mice. (**B**) Effect of HAE*Pa* on glycemia after a single oral administration of 2 g/kg of sucrose in male normoglycemic CD1 mice. Each plot represents the means + S.E.M. for six independent experiments. * *p* < 0.05 compared with control. (**C**) α-glucosidases activity inhibition of HAE*Pa* compared to hydroalcoholic extract from *Camelia sinensis* (green tea). The results represent the means ± S.E.M. for four independent experiments. * *p* < 0.05 compared with control.

HAE*Pa* was evaluated in glucose (OGTT) and sucrose (OSTT) tolerance curves, to corroborate its use as an antihyperglycemic agent. In Figure 6A, it is observed that HAE*Pa* (100 mg/kg) decreased (*p* < 0.05) the hyperglycemic peak produced by the glucose load delivered (2 g/kg) at 0.5 and 1 h after treatment, and the extract also diminished the entire area under the curve (AUC) of the group of animals treated vs. the control animals.

These results suggest that the effect might be related to a possible blockade of the intestinal glucose transporters, such as GLUT-2 and SGLT-1. Previously, it was described that verbascoside could suppress postprandial glucose concentrations by the inhibition of glucose transporter-1 at the intestinal level [28], which could be one of the responsible compounds for antihyperglycemic action in the OGTT.

Instead, the early decrease in the AUC, and the rapid establishment of baseline glucose values in treated animals, suggest a potential insulin-sensitizing action. For this purpose, some authors have demonstrated that other species of Plantago, such as *P. lanceolata*, *P. maxima*, *P. major* and *P. psyllium*, significantly decreased the hyperglycemic state, but they did not report any mechanisms of action [29,30,31,32,33]. However, UA, verbascoside and caffeic acid were widely described as insulin sensitizers with several mechanisms of action [28,34,35,36].

On the other hand, OSTT was also modified; moreover, the administration of 100 mg/kg of HAE*Pa* was more effective than the effect produced in OGTT, showing more effectiveness when diminishing the hyperglycemic peak, and significantly reduced the AUC (Figure 6B). This effect may possibly be related to the inhibition of α-glucosidases. These enzymes are responsible for hydrolyzing the bonds that hold the disaccharides and some polysaccharides together, to give rise to their corresponding monosaccharides so that they can be absorbed [37,38].

HAE*Pa* significantly decreased the %VG with respect to the control, with a similar pattern to acarbose (3 mg/kg) (Figure 6B). This experiment supports that the inhibition of α-glucosidases might be one of the antihyperglycemic mechanisms of action of the HAE*Pa*.

The inhibition of α-glucosidases by HAE*Pa* prevented sucrose hydrolysis, which resulted in less available glucose and delayed its transport, favoring the diminished hyperglycemic peak and the maintenance of postprandial glucose [37,38]. To test the last asseveration, we evaluated the effect of HAEP*a* (1 mg/mL) on α-glucosidases, showing a 52% inhibition of their activity (Figure 6C); the inhibitory effect seems moderate to account for the antidiabetic action, attributed to the species in traditional medicine. However, these results suggest that the compounds responsible for the antihyperglycemic effect are polar, given the nature of the extract. The compounds reported for *P. australis* are flavonoids, glycosylated iridoids, and phenolic and triterpenic compounds, among others [39], which could be responsible for the effect observed in OSTT. LC-MS experiments displayed the presence of caffeic acid (CA) for HAE*Pa,* and this hydroxycinnamic acid has been reported to significantly decrease glucose levels for healthy animals and could be contributing to the observed effect [24].

In this context, various authors suggested that some polar compounds, especially those that are glycosylated, possess an inhibitory activity for these enzymes since they are false substrates due to having α- or β-type bonds, which cannot be hydrolyzed by α-glucosidases [19,20]. One of these is verbascoside, which was previously described as a potent α-glucosidases inhibitor (0.5 mMol/L) [34].

#### 2.2.2. Acute Antidiabetic Assay

**Figure 7:**
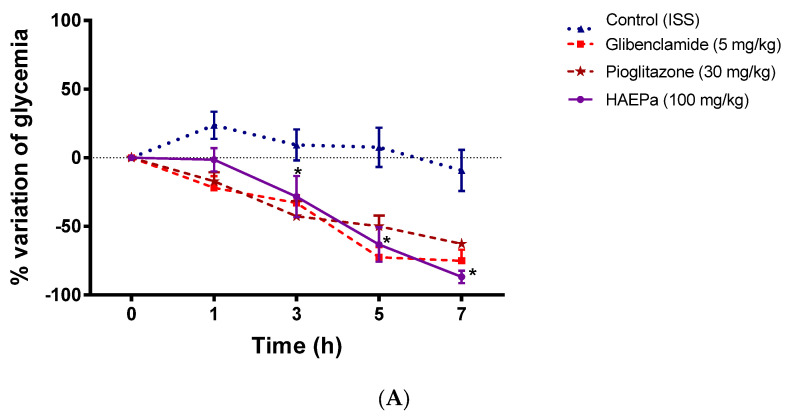

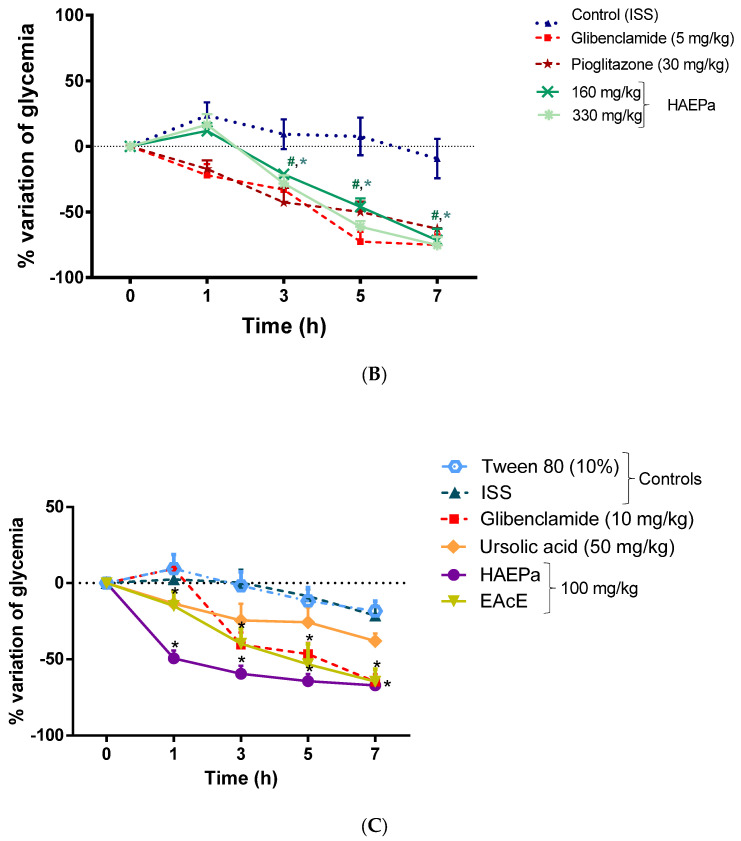
In vivo experimental non-insulin-dependent diabetes model. (**A**) Effect of a single dose (100 mg/kg) of HAE*Pa* in streptozotocin-nicotinamide-induced diabetes mice model. (**B**) Effect of multiple doses (160 and 330 mg/kg) of HAE*Pa*, in streptozotocin-nicotinamide-induced diabetes mice model. (**C**) Effect of extraction with ethyl acetate of the hydroalcoholic extract of *Plantago australis* (EAcE, 100 mg/kg) and UA (50 mg/kg) in streptozotocin-nicotinamide-induced diabetes mice model. Each plot represents the means ± S.E.M. for six independent experiments. #, * *p* < 0.05 compared with control.

To determine that *P. australis* has antidiabetic properties, HAE*Pa* was evaluated in an experimental non-insulin-dependent diabetes model, to observe if the extract shows acute antidiabetic effects at 100 mg/kg. Thus, the extract showed significant antidiabetic actions from hour 3, and the effect was maintained throughout the 7 h of the experiment (Figure 7A). Furthermore, the AUC for HAE*Pa* was comparable with that observed for pioglitazone, an insulin-sensitizer agent.

These results suggested that one mechanism of HAE*Pa* involved in the antidiabetic effect could be insulin sensitization [16]. In order to observe the influence of the dose on the antidiabetic effect, tests were carried out with higher doses (160 and 330 mg/kg), finding that the effect was not dose-dependent at the doses studied (Figure 7A,B). Perhaps this is the first report about the antihyperglycemic and antidiabetic effect of *Plantago australis*. However, several reports highlight the anti-inflammatory properties of various Plantago species, such as *P. lanceolata*, *P. major*, *P. erosa*, *P. altissima*, *P. reniformis* and *P. australis* [10,20,22,32,33], and the regulation of the inflammatory response might be involved in the HAE*Pa* insulin-sensitizing mechanisms. Moreover, Rodriguez-Moran et al. [20] reported that *P. psyllium* reduces glycemia, triglycerides and LDL cholesterol in patients with T2D, associated with intestinal mechanisms due to the high fiber content of this species.

On the other hand, based on the phytochemical analysis and with the purpose of relating it to bioactive compounds, UA was determined and quantified in the extract. As is known, UA was isolated from *P. australis* [14], which is a significant antidiabetic agent, mainly as a PTP-1B inhibitor [16]. However, we decided to determine the antidiabetic action of the organic phase obtained from the partitioning process (EAcE) and UA at doses of 100 and 50 mg/kg, respectively. As expected, both showed significant antidiabetic effects (Figure 7C), which may be related to the antidiabetic action revealed by the HAE*Pa* that could be linked to the presence of UA, previously reported for its antidiabetic properties [24]. Although UA is one of the bioactive compounds in the extract, it is necessary to conduct an exhaustive phytochemical study to find more bioactive antidiabetic compounds.

### 2.3. In Vitro Pharmacologic Assays: RNAm Expression of PPARγ and GLUT4

**Figure 8:**
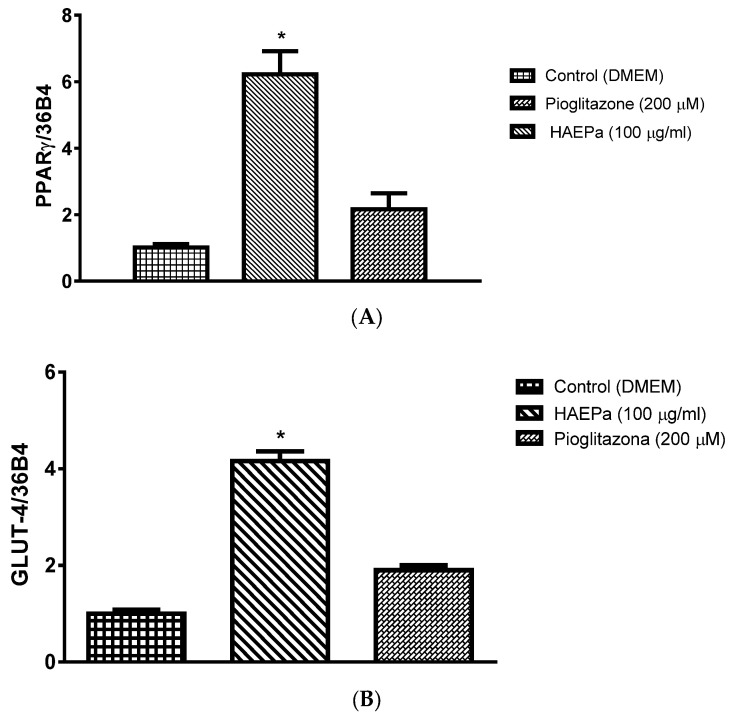
Effect of hydroalcoholic extract of Plantago australis (HAE*Pa*) on mRNA-induced expression of PPARγ (**A**) and GLUT4 (**B**). 3T3-L1 adipocytes cells were treated with HAEP*a* (100 µg/mL), and pioglitazone (PIO 5 µM) for 15 min. The results represent the means ± S.E.M. for six independent experiments. * *p* < 0.05 compared with control.

Based on the results obtained in the in vivo evaluations, we explored PPARγ and GLUT-4 expression as insulin-sensitizing mechanisms involved in the antidiabetic effect of HAE*Pa* and UA. The participation of this type of mechanism is important because, unlike the secretagogues, it does not deplete pancreatic β cells, nor does it produce hypoglycemia; however, associated weight gain has been linked [25]. The relative expression, induced by HAE*Pa*, of PPARγ mRNA in fibroblast 3T3-L1 differentiated to adipocytes was determined. As observed in Figure 8A, after 15 min. of treatment with HAE*Pa* (100 μg/mL), PPARγ expression increased ~sixfold vs. vehicle (*p* < 0.05). This result is a good indicator that this extract could be acting in this way, similar to the pattern observed in vivo tests. In addition, De Moura et al. [12] showed that an extract of *P. australis* standardized with verbascoside (phenolic glycoside compound), was anti-inflammatory in the LPS-induced inflammation in N9 microglial cells, where *P. australis* extract decreased proinflammatory cytokines TNF-α, IL-6 and IL-1β, as well as the NF-κB.

These findings and the presence of verbascoside in HAE*Pa* could be related to PPARγ activation, since its activation decreases the expression of proinflammatory cytokine genes (IL-6, TNF-α, IL-1β and IL-12) [40]. On the other hand, Volg et al. [41] carried out a study with more than 70 plant extracts to establish their anti-inflammatory potential; among these, *P. lanceolata* activated PPARs and inhibited NF-κB. This downregulation between PPARs and NF-κB could also be occurring with HAE*Pa*, since the activation of PPARγ inhibits NF-κB gene transcription, and this in turn positively regulates TNF-α and IL- 6 expression [42,43,44,45]. However, further experiments are necessary to corroborate the last asseveration.

**Figure 9:**
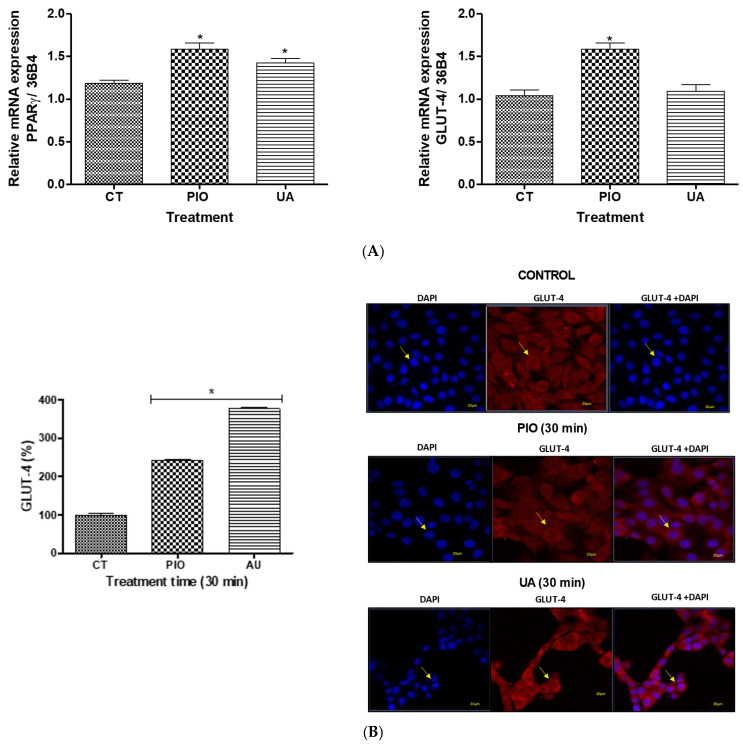
(**A**) Effect of UA on the mRNA expression of the PPARγ and GLUT4. C2C12 cells were treated with UA (10 µM), and pioglitazone (PIO 5 µM) for 15 min. Each evaluation represents the mean ± S.E.M. (n = 3). * *p* < 0.05 compared with control. (**B**) Translocation of GLUT4 after treatment of UA (10 µM) at 30 min. GLUT-4 content was then quantified at an OD of 510 nm and confocal fluorescence photomicrographs of GLUT-4 treated with UA (10 µM) for 30 min (magnification 40×). Each evaluation represents the mean ± S.E.M. (n = 3) * *p* < 0.05 compared with control; Yellow arrows indicate the GLUT-4 translocation.

Alternatively, several studies have demonstrated the anti-inflammatory effect of various species of Plantago, e.g., Palmeiro et al. [10] reported the anti-inflammatory action of *P. australis* in carrageenan-induced paw edema in rats; in this way, the regulation of the low-grade inflammatory response turns out to be a mechanism to improve insulin sensitization. To evaluate the impact of UA on another model with high metabolic activity that represents the cell target of many antidiabetic drugs, we selected the C2C12 cells; in these cells, UA significantly augmented the relative expression of PPARγ (Figure 9A) after treatment, and this was similar to pioglitazone, indicating that this triterpenic acid is one of the antidiabetic compounds responsible for HAE*Pa* being an insulin sensitizer.

On the other hand, HAE*Pa* (100 µg/mL) increased the GLUT-4 mRNA relative expression ~fourfold compared with the control (Figure 8B, *p* < 0.05). GLUT-4 overexpression is probably responsible for the decrease of glycemia in the in vivo tests carried out in the current investigation. These results agree with the results observed in Figure 8A, since PPARγ regulated the transcription of several genes involved in glucose metabolism, mostly the GLUT-4 transporter, the main glucose transporter activated by the action of insulin in muscle, adipose tissue, and liver. It occurs by subsequent phosphorylation that the crucial GLUT-4 translocation ends, which provokes glucose internalization into the cells of said tissues, resulting in its use [42,43,44,45]. However, UA (10 µM) did not increase the relative GLUT-4 overexpression (Figure 9A) but significantly produced the translocation of GLUT-4 in C2C12 cells (Figure 9B). Despite expression kinetics, the changes in GLUT-4 expression did not correlate in time with those of PPARγ. However, we consider that the most important event is the translocation of the transporter, since it reveals its availability for glucose uptake, which is associated with insulin sensitization as a antidiabetic mechanism. Future studies are needed to assess glucose uptake.

### 2.4. In Vivo Toxicological Studies

Medicinal plants are used by a large percentage of the population to treat a wide variety of diseases. However, only a few species have been studied to support their use and safety [46]. Therefore, in this project, the acute and sub-chronic toxicological study of HAEP*a* was carried out in accordance with the guidelines suggested by the OECD sections 423 and 407, respectively, with some modifications.

#### 2.4.1. Acute Toxic Class Method (LD_50_ Estimation)

In Table 1, the results of the acute toxicological study are shown, where no deaths were found at the doses after 24 h of treatment, and no apparent behavioral nor bodily changes were recorded after 14 days of observation. These results led us to classify HAE*Pa* according to the Globally Harmonized System of Classification and Labeling of chemical products (GHS) in category 4, establishing its LD_50_ as higher than 2000 mg/kg. In this context, Henn et al. [14] carried out a toxicological study with a standardized extract of *P. australis* (verbascoside 6%) and established its safety, since they did not find evidence of genotoxicity or mutagenicity and established its LD50 as > 5000 mg/kg without causing apparent toxicity.

#### 2.4.2. Sub-Chronic Toxicity Study

**Figure 10:**
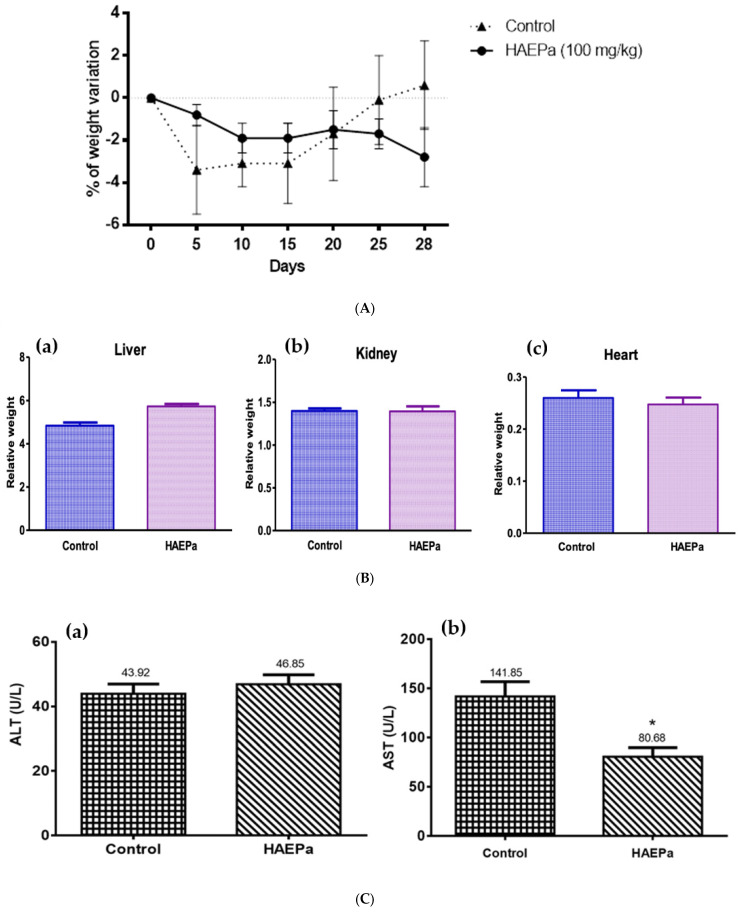
(**A**) Variation of weight of animal treated with HAE*Pa* (100 mg/kg) with respect to the control (vehicle) during 28 days of intragastric administration. Each plot represents the means + S.E.M. for ten independent experiments. (**B**) Relative weight of principal organs involved in the metabolism of exogenous agents: (**a**) liver, (**b**) kidney and (**c**) heart; after 28 days of intragastric administration. (**C**) (**a**) activity of amine alanine transferase (ALT) and (**b**) amine aspartate transferase (AST) after 28 days administration with HAE*Pa* (100 mg/kg), by intragastric route. The results represent the means ± S.E.M. for ten independent experiments. * *p* < 0.05 compared with control.

With the aim to observe the effect of the daily administration of HAE*Pa* (100 mg/kg) in the medium term, the weight of the mice was monitored during the 28-day period of treatment, where no significant changes were found compared with the control (Figure 10A).

Once the treated animals were sacrificed, the relative weight of the main organs involved in the metabolism of exogenous substances (liver and kidney) and the heart was determined, because various drugs or bioactive compounds can cause cardiotoxicity. In Figure 10B, no significant changes in the relative weight of any of the organs, with respect to the vehicle, can be observed, thus suggesting that there were no metabolic damages or apparent inflammatory processes.

Within this framework, the activity of ALT and AST, the main enzymes that indicate liver damage and damage to other organs, were also determined. For these, ALT is found predominantly in the liver parenchyma, while AST, in addition to the liver, is found in the myocardium, skeletal muscle, pancreas, and lungs. Both enzymes are found inside cells, but when an inflammatory process or injury occurs, they are released into the bloodstream, thus raising their plasma activity; however, the magnitude of this elevation does not correlate with its severity or extent and generally does not have a prognostic value [47,48]. Regarding the biochemical parameters associated with toxicity, in Figure 10C it can be seen that the ALT values show no significant changes with respect to the vehicle, and these were similar to the standard parameters (28–184 U/L) for mice of the CD1 strain, according to Kaneko [49].

Regarding the AST values (Figure 10C), the extract significantly decreased the plasma values with respect to the vehicle, even though these values again are in accordance with the standard parameters for this mouse strain (55–251 U/L). Therefore, data are not suggestive of any tissue injury. These results are similar to those described by Palmeiro et al. [50] for a hydroalcoholic extract of *P. australis*, which did not change ALT values; however, they also found that the AST values increased compared to the control at a higher dose (850 mg/kg). Moreover, the values of both enzymes were found within the standard parameters, so they were ruled out as indicative of damage. Furthermore, Henn et al. [14] found no significant changes in the ALT and AST of a standardized verbascoside extract of *P. australis* with respect to its vehicle.

**Figure 11:**
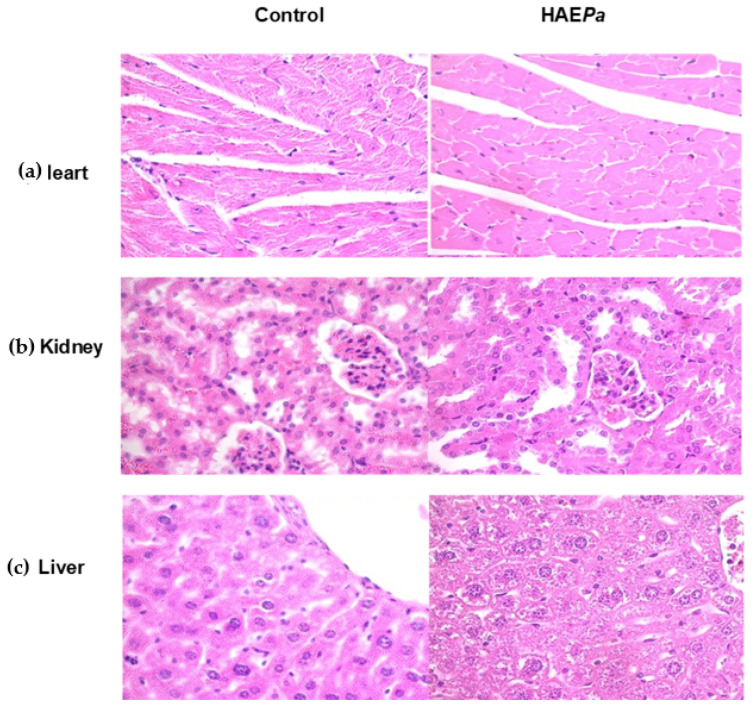
Photomicrographs of (**a**) Heart (**b**) Kidney (**c**) Liver the main metabolizing organs associated with toxicity with respect to control (vehicle) after 28 days of intragastric administration of HEA*Pa* (100 mg/kg).

A histological observation of the main metabolizing organs associated with toxicity (liver, kidney, and heart) was performed. In heart histology (Figure 11a), no apparent changes in myocytes and myocardial fibers were observed in the treated group. On the other hand, in the cyto-structure of the kidney (Figure 11b), there were no changes in the proximal and distal tubules, with adequate space in the Bowman’s capsule. Additionally, there was no presence of scaled cells or necrotic cells and no decrease in the number of nuclei in the group of treated animals compared to untreated animals.

Finally, in the liver (Figure 11c), we observed whole nuclei in the hepatocytes and normal centrilobular vein size in both groups, without the presence of necrotic cells or a decrease in the size of the central vein. Thus, these results provide evidence to confirm the absence of damage after treatment, for 28 days, with the hydroalcoholic extract of *P. australis* at the dose assayed. These results are added to and confirm the previous toxicity studies carried out on a hydroalcoholic extract of *P. australis* by Palmiero et al. [50] and that described by Henn et al. [14].

## 3. Materials and Methods

### 3.1. Chemicals and Drugs

For thin layer chromatography (TLC), sucrose, acarbose, nicotinamide, streptozotocin, pioglitazone, glibenclamide, ursolic acid, and aluminum sheets of silica gel coated with fluorescent indicator F_254_ 20 × 20 cm were purchased from Sigma-Aldrich Co. (St. Louis, MO, USA). Other reagents were analytical grade and acquired from local suppliers.

### 3.2. Plant Material Collection

To obtain the HAE*Pa* (hydroalcoholic extract of *P. australis*), the aerial parts of *Plantago australis* Lam. subsp. *hirtella* (Kurth) Rahn was used. Dr. Irene Perea-Arango (CEIB, UAEM) identified them from a collection in Tlalnepantla, state of Morelos, México, in July 2016 and stored them at the CIBIS Herbarium (HUMO Herbarium, UAEM). Voucher number 34,059 was assigned.

### 3.3. Preparation of the Extracts

The whole, dried aerial parts of *P. australis* (250 g) were subjected to extraction by maceration for 72 h (three times each) with water-ethanol (30:70). Then, the extract was filtered and concentrated with a rotary evaporator to obtain the dry extract, with 17.6% yield. As a strategy to fractionate the HAE*Pa,* a liquid–liquid extraction was performed with ethyl acetate. An extract sample (1.1 g) was dissolved in water (4 mL) and successively extracted (three times) with ethyl acetate (4 mL each) to obtain EAcE.

### 3.4. Phytochemical Study: Identification and Quantification of Ursolic Acid (UA) by HPLC Method

After EAcE was concentrated under reduced pressure, 0.11 g of a solid mixture precipitate was obtained. All extracts of HAE*Pa*, EAcE, and precipitate were observed by TLC, where characteristic pink spots were observed after acid oxidation; this could indicate the presence of pentacyclic terpenoid. Hence, extracts were compared with the UA standard to corroborate the possible presence of UA.

Chromatographic separation was performed using methanol/acidified water (85:15) with a Gemini column (4.6 × 75 mm) at a flow rate of 0.9 mL/min and a UV/Vis detector (Waters 2456) system at a wavelength of 250 nm. A solution of a known concentration of a commercial UA standard (Sigma-Aldrich, ≥97%) was injected in triplicate to identify the retention time of the peak. In addition, the UA contained in the HAE*Pa* and EAcE was quantified using a calibration curve. Seven solutions of the UA standard were injected at different concentrations, and the area under the curve of the peaks obtained in each injection was determined. Thus, the linear equation was obtained, with an r^2^ value of 0.99.

Once the linear equation was obtained, the HAE*Pa* and EAcE were injected in triplicate, and the AUC of each injection was calculated using the calibration curve; the data were extrapolated, and this allowed us to calculate the percentage of UA contained in the HAE*Pa*.

### 3.5. LC-MS Characterization

UPLC-ESI-MS characterization was performed using an ACQUITY UPLC H-Class Bio System (Waters^®^ Corp., Milford, MA, USA). The separation was conducted using an ACQUITY UPLC^®^ HSS T3 130 Å column (1.8 µm, 2.1 × 50 mm, Waters^®^ Corp., Milford, MA, USA) with a column temperature of 35 °C. For HAE*Pa,* we used an isocratic elution, using a binary system consisting of 20% ammonium hydroxide in water to 0.05% (A) and 80% acetonitrile (B); and for EAcE we used a binary system consisting of ammonium hydroxide in water to 0.05% (A) and acetonitrile (B). We used a gradient elution of 0–2 min 90% A, 2–4 min 80% A, 4–6 min 50% A, 6–8 min 20% A, and 8–9 min 90% A. Then, 5 μL of the samples and standards at 100 ppm concentration were injected with a flow rate of 0.4 mL/min, and methanol was used as blank solvent.

Detection was performed using an ACQUITY QDa detector mass spectrometer (Waters Corp., Milford, MA, USA) with an electrospray ionization interface (ESI); the voltage of the capillary was set to −1.0 kV for the negative-ion mode (ESI-). The data were processed using Waters Empower™ 3 software (Waters Corp., Milford, MA, USA). A mass scan acquisition was programmed at 50 to 1250 Da and a selected ion recording (SIR) for each targeted mass was selected [51].

### 3.6. In Vivo Pharmacologic Studies

#### 3.6.1. Animals

CD1 male mice (25–35 g) were used for both kinds of study; mice were kept at a constant room temperature and in a 12 h light/dark cycle. The experiments were carried out in accordance with the Federal Regulations for Animal Experimentation and Care (SAGARPA, NOM-062-ZOO-1999, Mexico) and approved by the Institutional Animal Care and Use Committee (Protocol 1857, U.A.M. Iztapalapa, Mexico), as well as international standards (approved by the Institutional Animal Care and Use Committee based on US National Institutes of Health Publication No.85–23, revised 1985) regarding the care and use for experimental animals. Mice were fed ad libitum with a standard diet and water, except when fasting was needed during the study. For each experimental procedure, groups consisted of six mice. For the acute toxicological study, mice were allocated to groups of three.

#### 3.6.2. Oral Glucose or Sucrose Tolerance Tests

Normoglycemic animals were separated in three groups (n = 6):

Group 1: test sample (HAEP*a*, 100 mg/kg).

Group 2: control (isotonic saline solution, ISS).

Groups 3: positive control (glibenclamide, 5 mg/kg or acarbose, 3 mg/kg).

A load of 2 g/kg of glucose or sucrose solution was administered to mice 30 min after test samples. Then blood samples were obtained at time 0 (before oral administration), 0.5, 1, 1.5, 2, and 3 h after the vehicle, positive control, and extract administrations, from the caudal vein. Glycemia was estimated by the glucose dehydrogenase method using a commercial glucometer (Accu-Chek, Performa; Roche^®^). The percentage change of glycemia for each group was calculated in relation to the initial (0 h) level, according to the formula:

%Variation of glycemia = [(Gx − G0)/G0] × 100

where G0 is the initial glycemia value and Gx is the glycemia value at each time point [16].

#### 3.6.3. Induction of Diabetes

A non-insulin-dependent diabetic mice model was obtained as described [52,53]. Briefly, mice were administered with a single intraperitoneal (i.p.) injection with streptozotocin (120 mg/kg) dissolved in a citrate buffer (pH = 4.5), 15 min after injection of nicotinamide (40 mg/kg) dissolved in distilled water. Hyperglycemia was confirmed one week later by glycemia over 180 mg/dL, measured with a glucometer (Accu-Chek^®^).

#### 3.6.4. Acute Antidiabetic Assay

Diabetic mice were randomly divided into six groups (n = 6):

Group 1: vehicle (isotonic saline solution).

Group 2: glibenclamide (5 mg/kg).

Group 3: pioglitazone (30 mg/kg).

Groups 4, 5 and 6: HAE*Pa* (100, 160 and 330 mg/kg, respectively).

Group 7: EAcE (100 mg/kg).

Group 8: Ursolic acid (50 mg/kg).

Blood samples were collected from the caudal vein at 0 time before treatments, and at 1, 3, 5, and 7 h after the administration of the vehicle, test samples and positive control. Glycemia was estimated as described [53].

### 3.7. In Vitro Pharmacologic Assays

#### 3.7.1. mRNA Expression Analysis of PPARγ and GLUT

To analyze the effects over two cellular targets with high metabolic activity, we selected adipocyte (3T3-L1) to evaluate the general effect, and muscular cells (C2C12) to evaluate the specific effect of one of the most important components of *P. australis*.

Moreover, 3T3-L1 murine fibroblasts (9 × 10^−5^ cells per well) (CL-173; Lot number: 70032508. ATCC, American Type Culture Collection, Manassas, VA, USA) were cultured in 75 cm^2^ bottles (Corning Incorporated, NY, USA) in Dulbecco’s Modified Eagle’s Medium (DMEM) supplemented with, 25 mM glucose, 10% fetal bovine serum (*v*/*v*), 1 mM sodium pyruvate, 2 mM glutamine, 0.1 mM non-essential amino acids, and gentamicin, in a 5% CO_2_ humidified atmosphere, at 37 °C. The culture was maintained under standard growing conditions and the growth medium was changed every two days.

The HAE*Pa* effect on PPARγ and GLUT4 expression was determined in fibroblasts 3T3-L1 according to Chávez-Silva et al. [54]. The 3T3-L1 cells (~80% confluence) were differentiated to the adipocyte phenotype with a mix for differentiation (0.5 μM 3-isobutyl-1-methylxanthine, 0.25 μM dexamethasone acetate, and 0.8 μM insulin for 48 h), followed by insulin for 48 h more. The culture medium without insulin was changed every two days during eight days of differentiation.

C2C12 muscle cells (CRL-1772; Lot number: 70026471. ATCC, American Type Culture Collection, Manassas, VA, USA) were cultured in 75 cm^2^ bottles (Corning Incorporated, NY, USA) in medium DMEM (Dulbecco’s Modified Eagle’s Medium) supplemented with 25 mM glucose, 1 mM sodium pyruvate, 0.1 mM nonessential amino acids, 1% gentamicin, and 10% FBS. The culture was maintained under standard growing conditions and the growth medium was changed every two days. The UA effect on PPARγ and GLUT4 expression and GLUT4 translocation was evaluated in C2C12 myoblasts as described by Giacoman-Martínez et al. [55]. C2C12 cells were cultured and maintained by changing the medium every two days.

Later, the effect of HAEP*a* or UA on PPARγ and GLUT-4 expression was determined, and the cells were treated with the HAE*Pa* (100 μg/mL) or UA (10 µM) for 15 min [this time was determined by expression kinetics]. The mRNA was isolated using trizol (TriPure isolation reagent, Invitrogen) and total RNA was reverse-transcripted; the reaction was incubated in a thermocycler following the cycle program: incubation (25 °C for 5 min), extension (42 °C for 55 min). The enzyme was inactivated at 70 °C for 15 min. The reverse transcriptase reaction was amplified with an SYBR Green master mix (Roche Molecular Biochemicals, Mannheim, Germany) containing 0.5 μM of customized primers:

36B4, F-AAGCGCGTCCTGGCATTGTCT; R-CCGCAGGGGCAGCAGTGGT (Gene Bank Gene Bank NM_007475.2). PPARγ, F-CCAGAGTCTGCTGATCT GCG; R-GCCACCTCTTTGCTCTGCTC (Gene Bank NM_011146.1). GLUT4, F-GATTCTGCTGCCCTTCTGTC; R-ATTGGACGCTCTCTCTCCAA (Gene Bank NM_009204.2).

PCR was conducted using the following cycling conditions: pre-incubation and denaturation (95 °C/10 min). Amplification for 35 or 40 cycles that included: denaturation (95 °C/10 s) with a thermal ramp (20 °C/s); annealing (61 °C/7 s); amplification (72 °C/10 s). The threshold cycles (Ct) were measured in separate tubes (quadruplicate). The melting curve was analyzed at the end of the amplification following SYBER Green kit conditions, as indicated by the company (Roche Molecular Biochemicals).

Relative changes in the expression level of one specific gene (ΔΔCt) were calculated as ΔCt of the test group minus ΔCt of the control group, and then presented as 2_ΔΔCt.

#### 3.7.2. Glut GLUT4 Translocation

The C2C12 myoblasts were grown using the Chamber Slide System (Lab-Tek II, Thermo-Fisher, Waltham, MA, USA). After confluence, the cells were incubated with 10 µM UA for 30 min. After incubation, cells were evaluated according to Loza-Rodríguez et al. [20]. for the immunodetection of GLUT4 translocation. Primary GLUT4 antibody (Santa Cruz Biotechnology, Dallas, TX, USA) and secondary antibody (anti-rabbit-rhodamine; Santa Cruz Biotechnology, TX, USA) were used. Subsequently, an assembly of PBS/glycerol/DAPI (Inorganic Polyphosphate Stores by 4′,6-diamidino-2-phenyl-indole) (Abcam, ab228549) was used as a staining reagent for DNA detection and nuclear integrity. Cell images were taken with rhodamine-labeled GLUT4 in a Zen-Sp1 ZEISS confocal microscope [56]. Five fields were randomly selected in each well, and the average pixel intensity was measured using the ImageJ program (Bethesda, MD, USA).

#### 3.7.3. α-Glucosidases Inhibition

A described protocol was used [57]. Starch (12.5 mg/mL), an α-glucosidase enzymes complex (obtained from intestinal brush border of Wistar rats), and HAE*Pa* (1 mg/mL) were added to the reaction tube, which were incubated at 37 °C for 10 min. *Camellia sinensis* (1 mg/mL, HAE*Cs*) was used as a positive control (α-glucosidases inhibitor). The released glucose was quantified by a glucose oxidase-based clinical reagent (SPINREACT, Girona, Spain^®^) following the manufacturer’s instructions.

### 3.8. In Vivo Toxicological Studies

#### 3.8.1. Acute Toxic Class Method (LD50 Estimation)

The LD_50_ of the active extract of HAE*Pa* was established following OECD guide 423 with modifications. Male mice were used, and the doses (5, 50, 300 and 2000 mg/kg) were tested in different groups (four groups of three mice each). At the end of the study, the LD_50_ range (category) was established and categorized using the Globally Harmonized Classification System (GHS).

#### 3.8.2. Sub-Chronic Toxicity Study

The protocol used was established following OECD guide 407 with modifications. Two groups of male mice were formed (n = 8):

Group 1: vehicle (isotonic saline solution).

Group 2: treatment (HAE*Pa*, 100 mg/kg).

Both groups of mice were administered over 28 days under the same conditions and were monitored daily in both body and behavior. After the experiment (day 29), each mouse was bled by cardiac puncture to determine biochemical parameters such as Aspartate Aminotransferase (AST) and Alanine Aminotransferase (ALT) activities, to evaluate toxicity.

On the other hand, the relative organ weight (ROW) of both experimental groups was determined with the following equation:

ROW (%) = (Organ weight/final weight of each mouse) × 100

Finally, the main metabolizing organs (liver, kidney, and heart) were processed to carry out histological analyses. The presence or absence of morphological cell alterations were compared between the treated and control groups. The organs were maintained in a 10% saline buffer and embedded in paraffin. The paraffin embedded tissues were cut into 4 mm sections with a microtome and stained with hematoxylin and eosin (HandE) for the analyses. Histological slides were examined under an upright Zeiss Axios kop with objectives: 10×, 20× and 40×.

### 3.9. Results Presentation and Statistical Analysis

All values are expressed as the mean ± S.E.M. for in vivo (six mice per group) or in vitro (cells in sextuplicate) studies. Analysis of variance (ANOVA) was used to analyze changes in the percentage variation of glycemia, followed by Bonferroni post-tests; for in vitro assays, ANOVA was used followed by Dunnett’s multiple comparison test; *p* < 0.05 was considered statistically significant. GraphPad Prisma software was used for data analyses.

## 4. Conclusions

The hydroalcoholic extract of *P. australis* (HAE*Pa* 100 mg/kg) showed significant antihyperglycemic and antidiabetic effects, possibly through extra-pancreatic mechanisms of action, which involve the inhibition of α-glucosidases and the overexpression of PPARγ and GLUT-4. This also suggests that the regulation of the low-grade inflammatory response is associated with T2D, and other concomitant conditions. UA is one of the antidiabetic compounds present in the extract that induces the overexpression of PPARγ and GLUT-4 translocation; however, the presence of caffeic acid, verbascoside and geniposidic acid increased the antidiabetic activity of the HAE*Pa*. On the other hand, current data add to those described on the lack of *P. australis* toxicity, and they allow us to establish the safety of the development and use of the phytopharmaceutical content of the hydroalcoholic extract from this species.

## Figures and Tables

**Table 1:** Results of acute toxicity of the hydroalcoholic extract of *Plantago australis* test the according to the OECD.

Doses of HAE*Pa* (mg/kg)	Deaths	DL_50_	Classification (GHS)
5	0	>2000 mg/kg	Category 4
50	0		
300	0		
2000	0		

## Data Availability

Data is contained within the article and Supplementary Material.

## Supplementary Materials

The following supporting information can be downloaded at: https://www.mdpi.com/article/10.3390/ph16040535/s1, Figure S1: Comparative TLC comparison for standard compounds and extracts from *Plantago australis*; Figure S2: (A) 1H NMR spectra 400 MHz (B),D overlayed 1H, 13C NMR EAcE spectra with reference (C) 13C NMR spectra 100 MHz, EAcE from *Plantago australis*; Figure S3: Total Ion Chromatogram (TIC) for HAE*Pa* exploratory mass scan from 10–1000 Da [the x-axis represents time, and y-axis represents signal intensity], and 3D Chromatogram; Figure S4: Comparative Selected Ion Recording (SIR) for (a) HAEPa (b) Verbascoside standard (c) Methanol channel selected for 623.60 Da in negative mode (ESI-); Figure S5: Comparative SIR for (a) HAEPa (b) caffeic acid standard (c) Methanol channel selected for 179.16 Da in negative mode (ESI-); Figure S6: Comparative SIR for geniposidic acid identification (a) HAE*Pa* (b) Methanol channel selected for 373.34 Da in negative mode (ESI-); Figure S7: Stack plot for EAcE, first Total Ion Chromatogram (TIC) for EAcE exploratory mass scan from 100–1000 Da [the x-axis represents time, and y-axis represents signal intensity], and 3D Chromatogram contrasted in second section with selected channel from mass scan at 455.7 Da m/z for UA identification, peak 1 retention time 7.025, mass detected 455.67, 456.66 Da m/z [M-1], peak 2 retention time 7.247, mass detected 455.70, 456.65 Da m/z [M-1] acquired in negative mode [ESI-]; Figure S8: Bioethical Commission Acceptance.
