# Supplementary material for: Insulin Sensitization by PPARγ and GLUT-4 Overexpression/Translocation Mediates the Antidiabetic Effect of Plantago australis
Source: Pharmaceuticals (Basel). 2023 Apr 3;16(4):535. doi: 10.3390/ph16040535 (PMC10143998; doi:10.3390/ph16040535)
Supplement: Supplementary file 1 [file pharmaceuticals-16-00535-s001.zip › pharmaceuticals-2242105-supplementary.pdf]

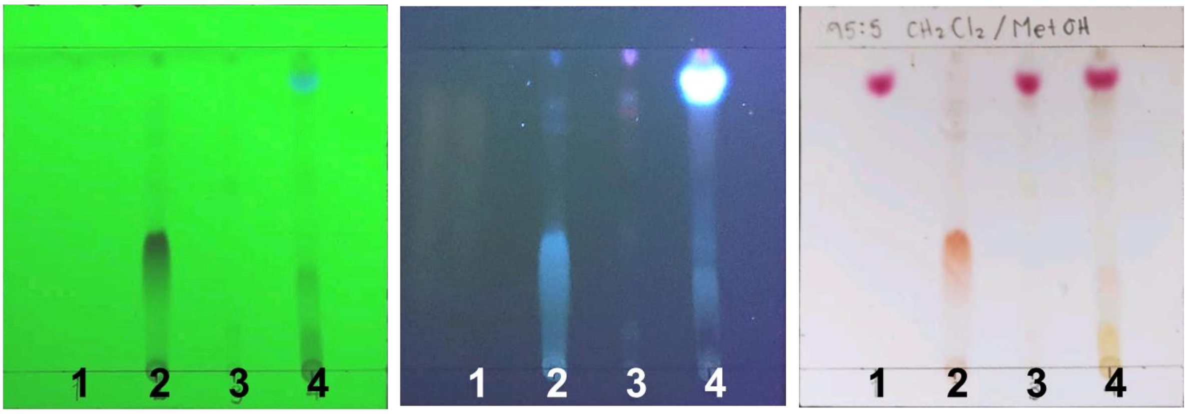

Mobil phase: 95:5 CH_2Cl_2- methanol 1) UA 2) Verbascoside 3) EAcE 4) HAE*Pa*, observed in λ=254 nm, λ = 365 nm and visible light oxidated with sulphuric acid 10% respectively.

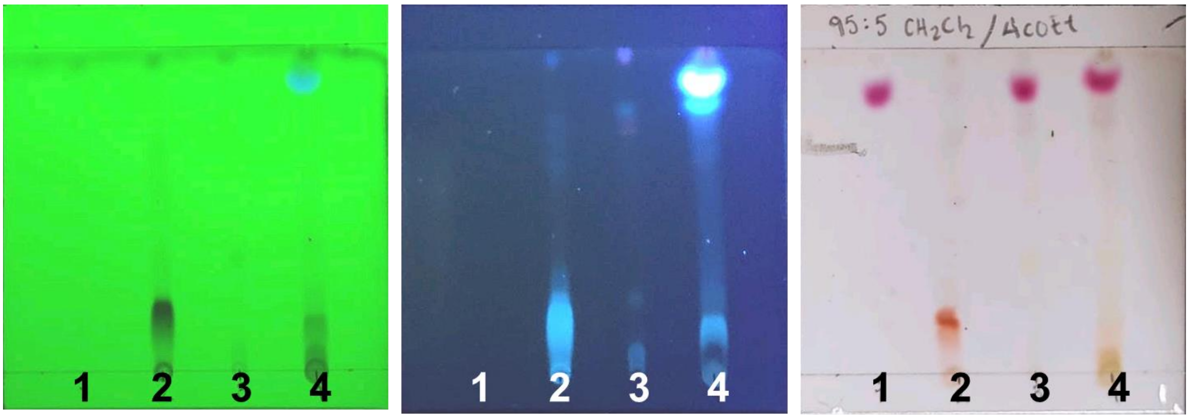

Mobil phase: 95:5 CH_2Cl_2-Ethyl Acetate 1) UA 2) Verbascoside 3) EAcE 4) HAE*Pa*, observed in l=254 nm, l = 365 nm and visible light oxidated with sulphuric acid 10% respectively.

Figure S1: Comparative TLC comparison for standard compounds and extracts from *Plantago australis.*

(A)

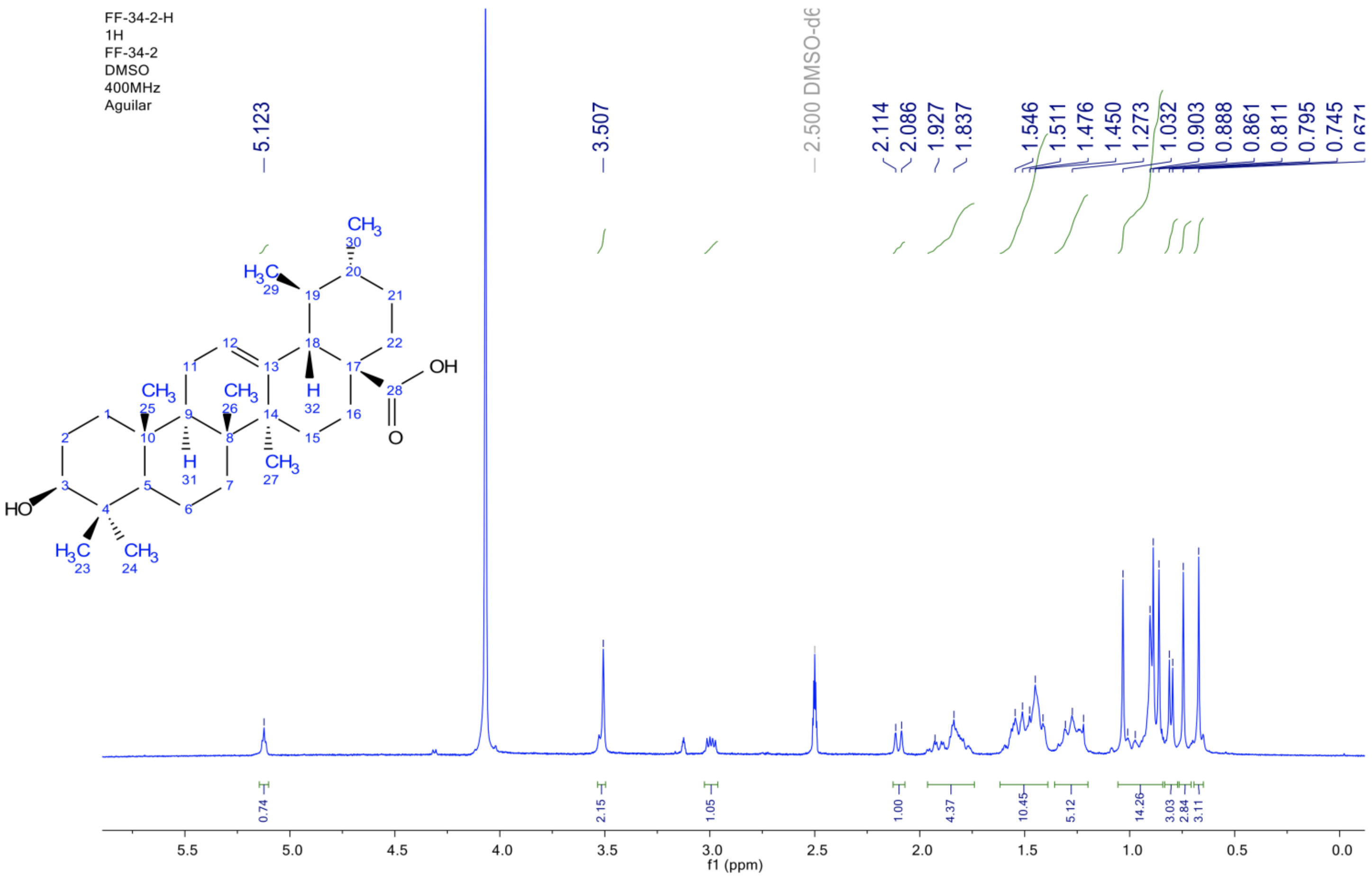
FF-34-2-H
1H
FF-34-2
DMSO
400MHz
Aguilar
5.123
3.507
2.500 DMSO-d6
2.114
2.086
1.927
1.837
1.546
1.511
1.476
1.450
1.273
1.032
0.903
0.888
0.861
0.811
0.795
0.745
0.74
2.15
1.05
1.00
4.37
10.45
5.12
14.26
3.03
2.84
3.11
5.5
5.0
4.5
4.0
3.5
3.0
2.5
2.0
1.5
1.0
0.5
0.0
f1 (ppm)
HO
OH
O

(B)

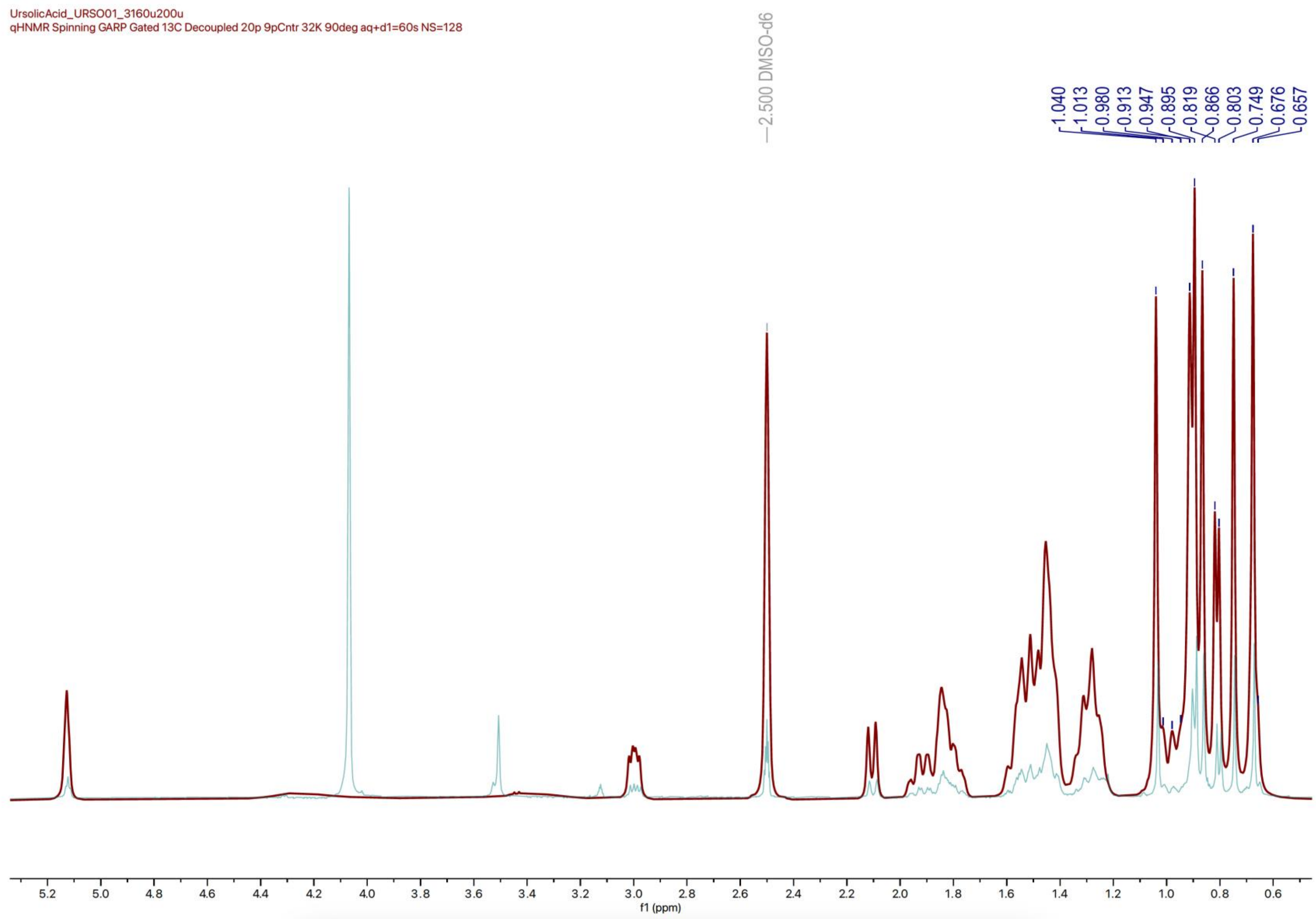
UrsolicAcid_URSO01_3160u200u
qHNMR Spinning GARP Gated 13C Decoupled 20p 9pCntr 32K 90deg aq+d1=60s NS=128
2.500 DMSO-d6
1.040
1.013
0.980
0.913
0.947
0.895
0.819
0.866
0.803
0.749
0.676
0.657
5.2
5.0
4.8
4.6
4.4
4.2
4.0
3.8
3.6
3.4
3.2
3.0
2.8
2.6
2.4
2.2
2.0
1.8
1.6
1.4
1.2
1.0
0.8
0.6
f1 (ppm)

(C)

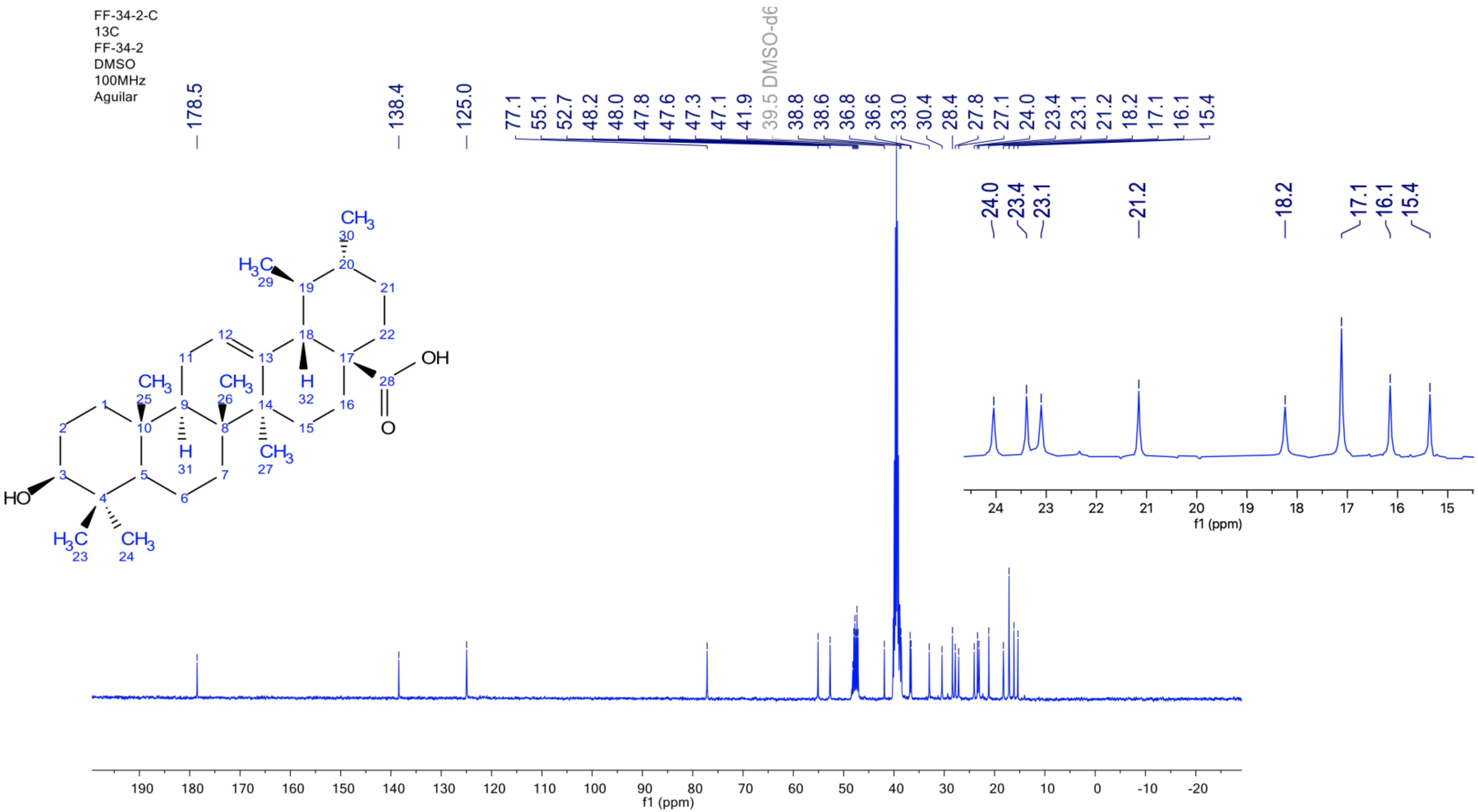
FF-34-2-C
13C
FF-34-2
DMSO
100MHz
Aguilar
178.5
138.4
125.0
77.1
55.1
52.7
48.2
48.0
47.8
47.6
47.3
47.1
41.9
39.5 DMSO-d6
38.8
38.6
36.8
36.6
33.0
30.4
28.4
27.8
27.1
24.0
23.4
23.1
21.2
18.2
17.1
16.1
15.4
HO
OH
O
f1 (ppm)
f1 (ppm)

(D)

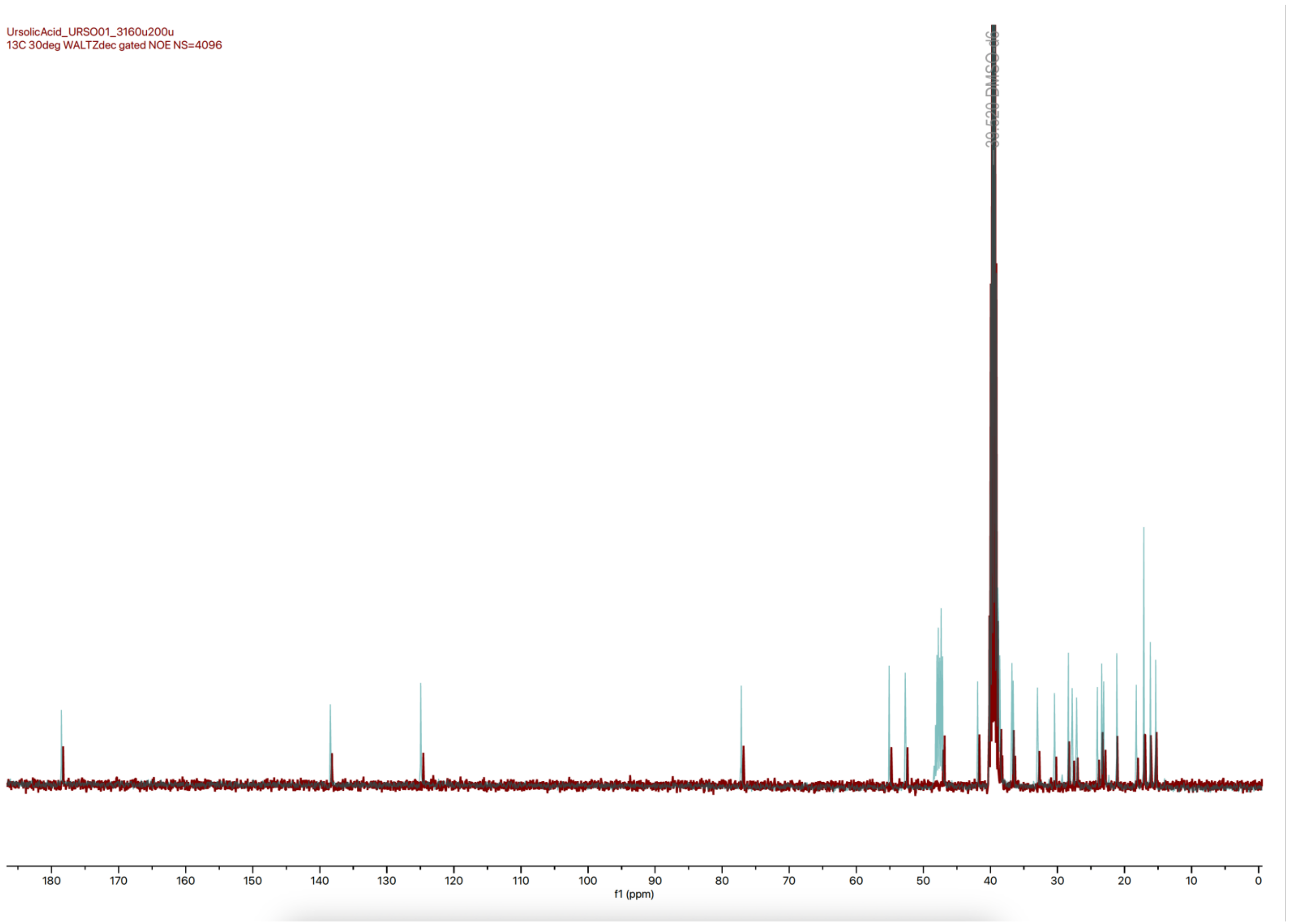

Figure S2: (**A**) 1H NMR spectra 400 MHz (**B**,**D**), overlayed 1H, 13C NMR EAcE spectra with reference (**C**) 13C NMR spectra 100MHz, EAcE from *Plantago australis* [57].

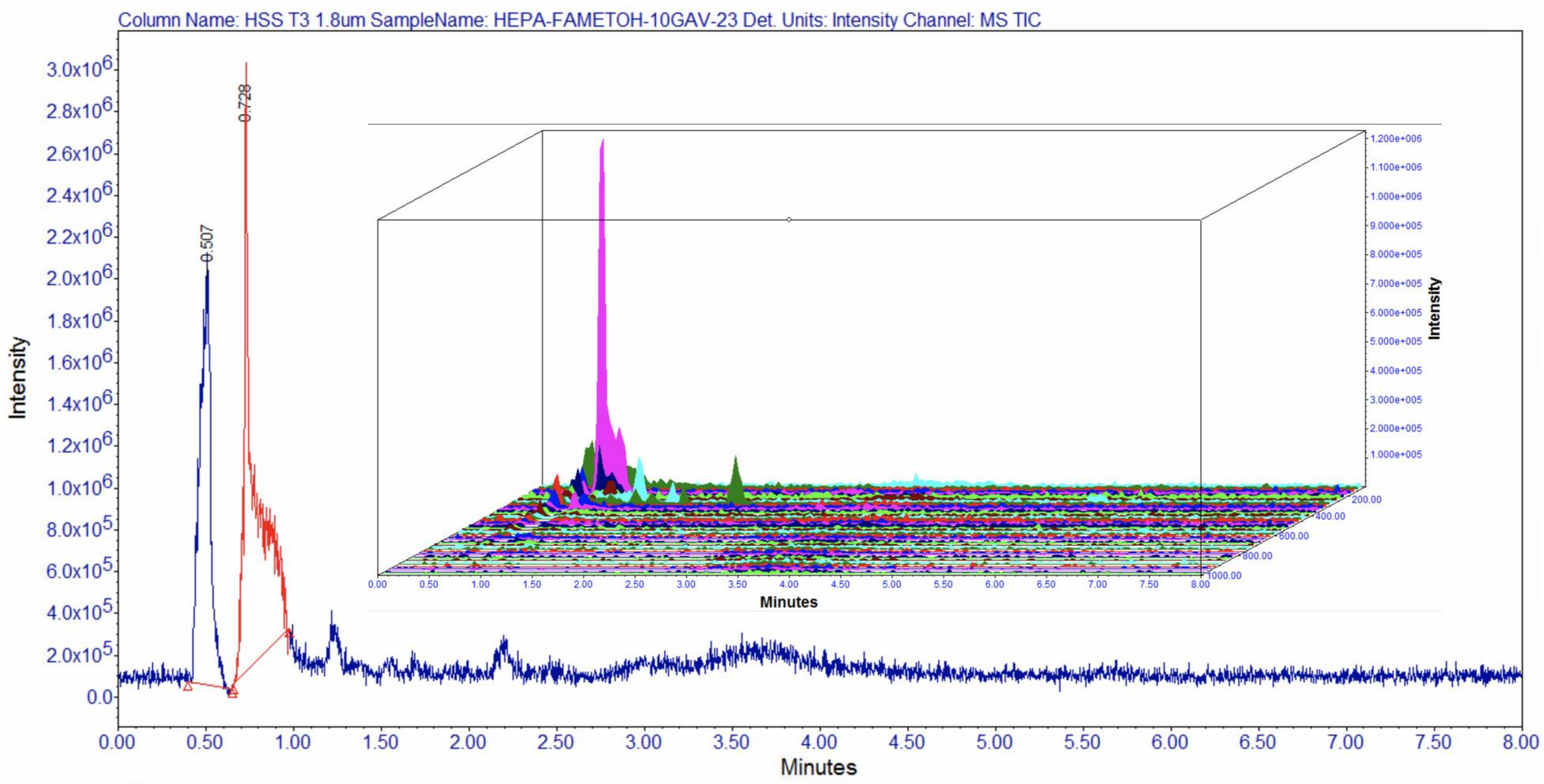

Figure S3: Total Ion Chromatogram (TIC) for HAE*Pa* exploratory mass scan from 10–1000 Da [the x-axis represents time, and y-axis represents signal intensity], and 3D Chromatogram.

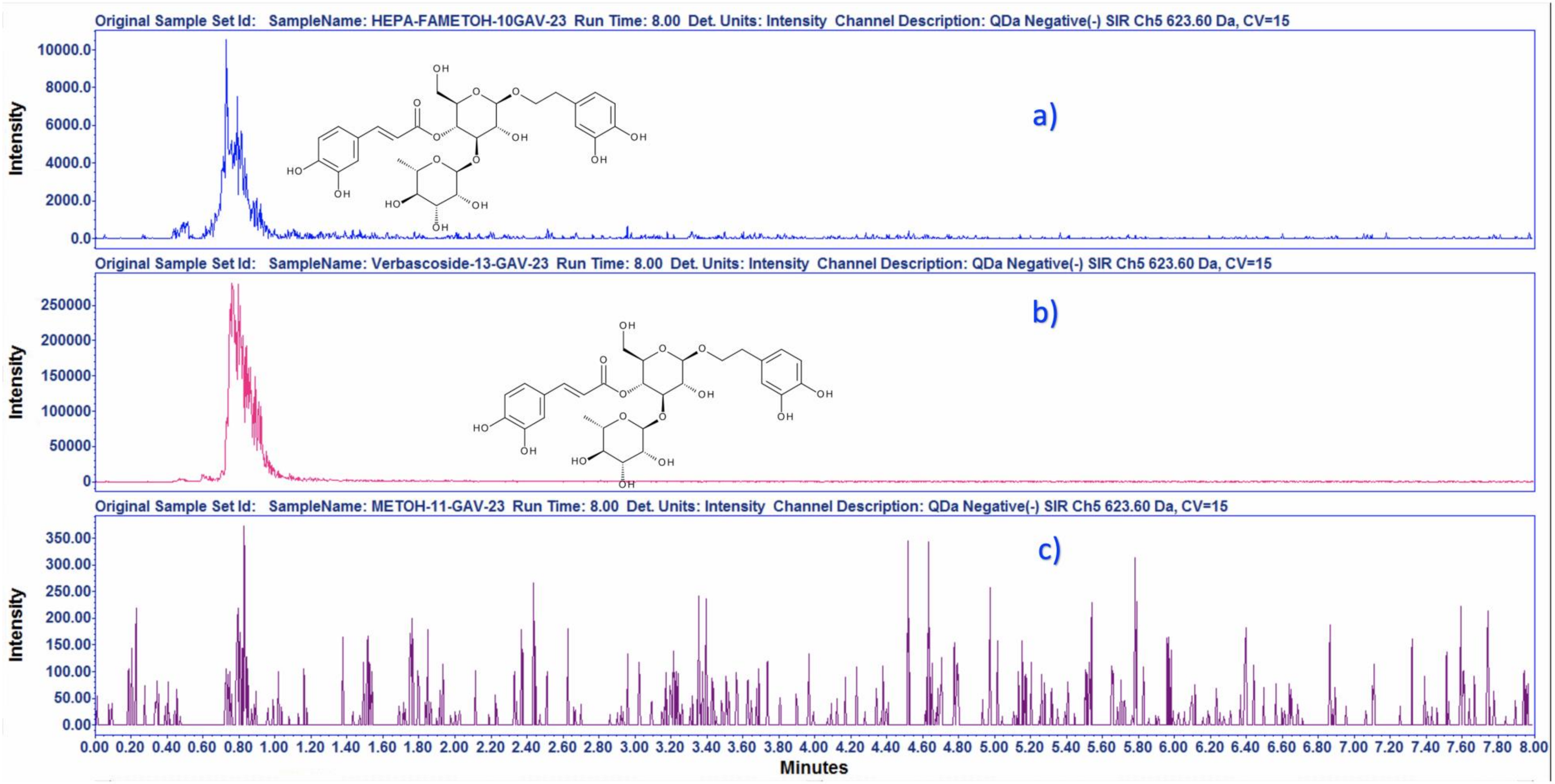

Figure S4: Comparative Selected Ion Recording (SIR) for (**a**) HAE*Pa* (**b**) Verbascoside standard (**c**) Methanol channel selected for 623.60 Da in negative mode (ESI-).

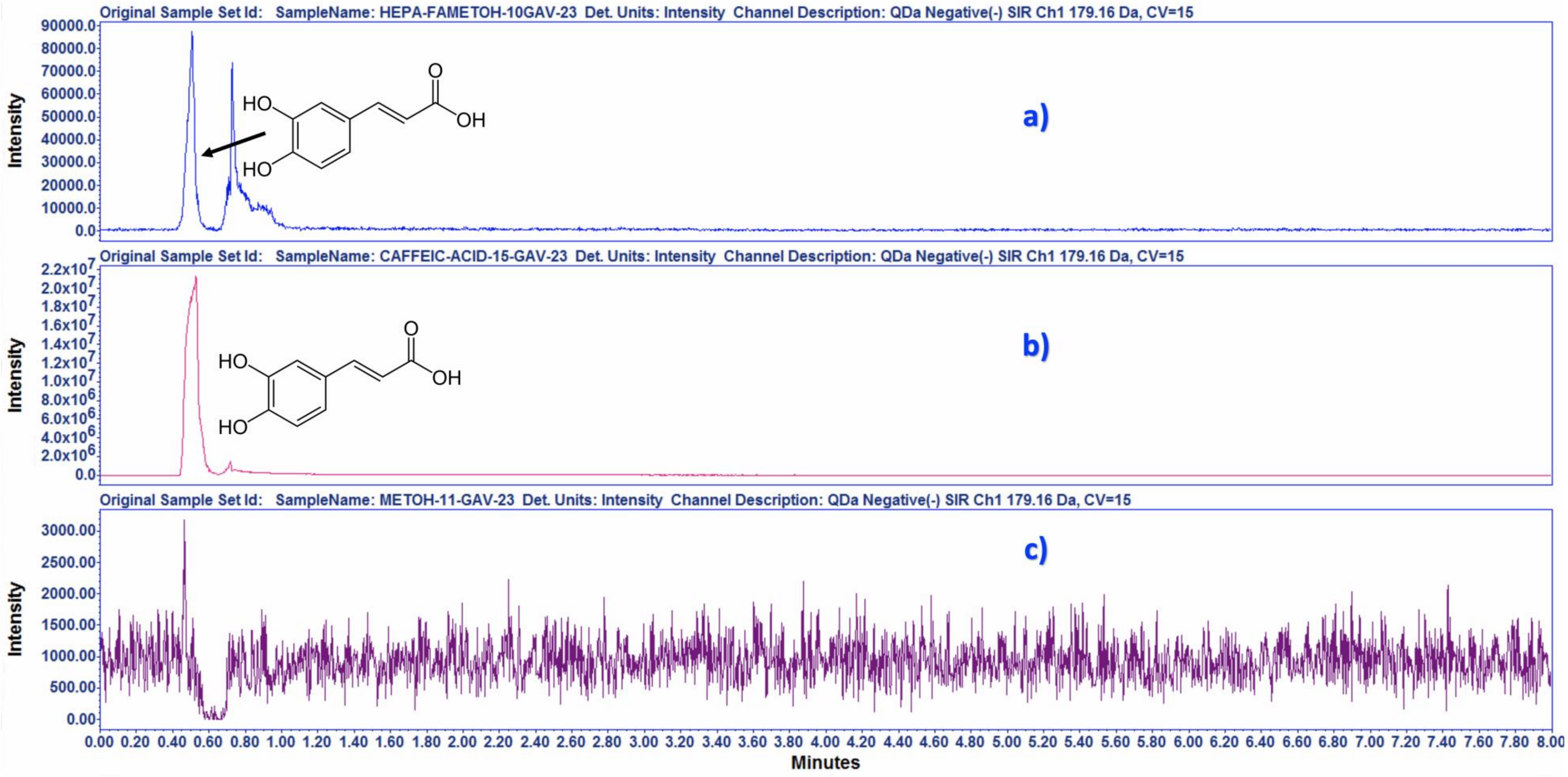

Figure S5: Comparative SIR for (**a**) HAE*Pa* (**b**) caffeic acid standard (**c**) Methanol channel selected for 179.16 Da in negative mode (ESI-).

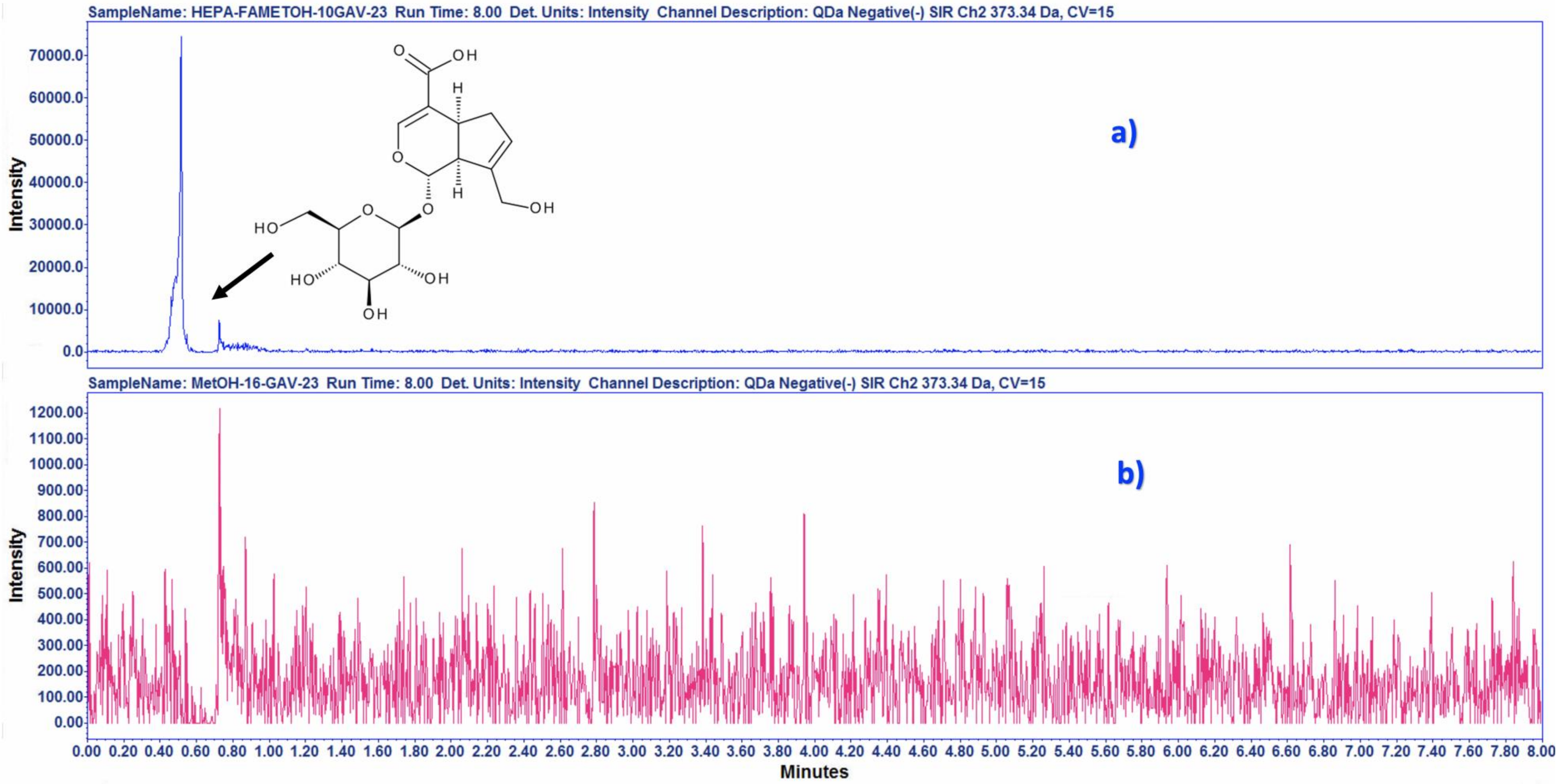

Figure S6: Comparative SIR for geniposidic acid identification (**a**) HAE*Pa* (**b**) Methanol channel selected for 373.34 Da in negative mode (ESI-).

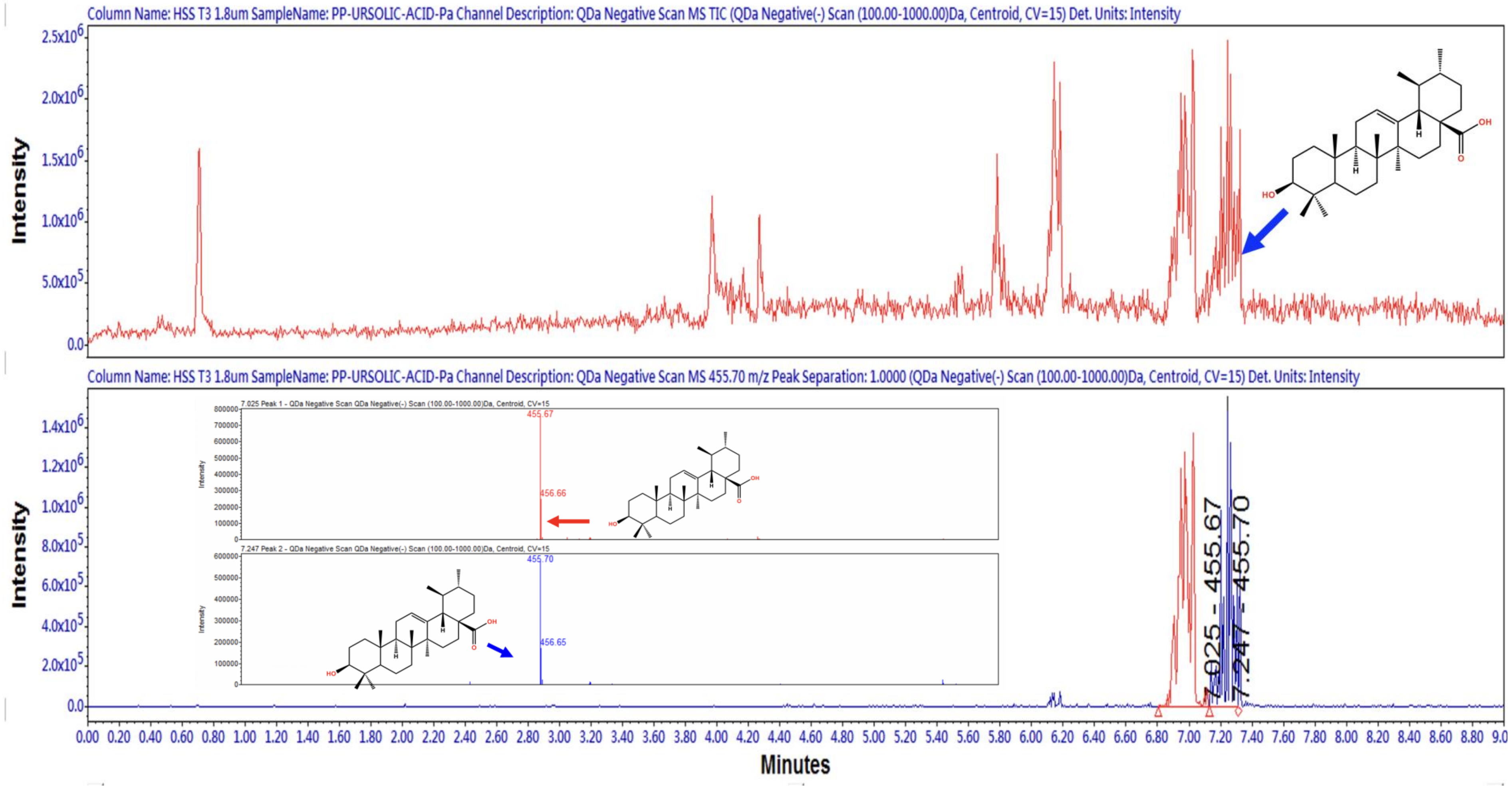

Figure S7: Stack plot for EAcE, first Total Ion Chromatogram (TIC) for EAcE exploratory mass scan from 100–1000 Da [the x-axis represents time, and y-axis represents signal intensity], and 3D Chromatogram contrasted in second section with selected channel from mass scan at 455.7 Da m/z for UA identification, peak 1 retention time 7.025, mass detected 455.67, 456.66 Da m/z [M-1], peak 2 retention time 7.247, mass detected 455.70, 456.65 Da m/z [M-1] acquired in negative mode [ESI-].

Casa abierta al tiempo

UNIVERSIDAD AUTÓNOMA METROPOLITANA-*Iztapalapa*
COMISIÓN ACADÉMICA DE ETICA DE LA DIVISIÓN DE CIENCIAS BIOLÓGICAS Y DE LA SALUD

Ciudad de México a 31 de enero de 2019
Dictamen: 1857

A quien corresponda:

La Comisión Académica de Ética de la División de Ciencias Biológicas y de la Salud, conforme a sus competencias revisó el protocolo de Investigación titulado: **"FARMACOLOGÍA Y QUÍMICA DE SUSTANCIAS PARA EL TRATAMIENTO DEL SÍNDROME METABÓLICO Y OTRAS ENFERMEDADES CRÓNICO DEGENERATIVAS"** a cargo del **Dr. Rubén Román Ramos.**

Encontrando que dicho proyecto, **SI CUMPLE CON LOS LINEAMIENTOS PARA LA CONDUCCIÓN ÉTCA DE LA INVESTIGACIÓN, LA DOCENCIA Y LA DIFUSIÓN EN LA DIVISIÓN DE CIENCIAS BIOLÓGICAS Y DE LA SALUD DE ESTA INSTITUCIÓN.**

Cabe recordar que dichos lineamientos derivan de la legislación mexicana que atañe a la parte bioética de nuestro quehacer.

Se extiende la presente para los fines que convengan al interesado

ATENTAMENTE
Casa Abierta al Tiempo

Dra. Ma. del Rosario Tarragó Castellanos
Presidenta

c.c.p. archivo

UNIDAD IZTAPALAPA Av. San Rafael Atlixco 186 Col. Vicentina. C.P. 09340, Ciudad de México

Figure S8: Bioethical Commission Acceptance.
